# Antibiotic-mediated dysbiosis leads to activation of inflammatory pathways

**DOI:** 10.3389/fimmu.2024.1493991

**Published:** 2025-01-09

**Authors:** Jemma J. Taitz, Jian Tan, Duan Ni, Camille Potier-Villette, Georges Grau, Ralph Nanan, Laurence Macia

**Affiliations:** ^1^ Charles Perkins Centre, The University of Sydney, Sydney, NSW, Australia; ^2^ School of Medical Sciences, Faculty of Medicine and Health, The University of Sydney, Sydney, NSW, Australia; ^3^ Vascular Immunology Unit, Discipline of Pathology, School of Medical Sciences, University of Sydney, Sydney, NSW, Australia; ^4^ Sydney Medical School Nepean, The University of Sydney, Sydney, NSW, Australia

**Keywords:** gut microbiota, dysbiosis, antibiotics, vancomycin, Polymyxin B, TNF, IBD, autoimmunity

## Abstract

**Introduction:**

The gut microbiota plays a pivotal role in influencing host health, through the production of metabolites and other key signalling molecules. While the impact of specific metabolites or taxa on host cells is well-documented, the broader impact of a disrupted microbiota on immune homeostasis is less understood, which is particularly important in the context of the increasing overuse of antibiotics.

**Methods:**

Female C57BL/6 mice were gavaged twice daily for four weeks with Vancomycin, Polymyxin B, or PBS (control). Caecal microbiota composition was assessed via 16S rRNA sequencing and caecal metabolites were quantified with NMR spectroscopy. Immune profiles of spleen and mesenteric lymph nodes (MLNs) were assessed by flow cytometry, and splenocytes assessed for *ex vivo* cytokine production. A generalised additive model approach was used to examine the relationship between global antibiotic consumption and IBD incidence.

**Results:**

Antibiotics significantly altered gut microbiota composition, reducing alpha-diversity. Acetate and butyrate were significantly reduced in antibiotic groups, while propionate and succinate increased in Vancomycin and PmB-treated mice, respectively. The MLNs and spleen showed changes only to DC numbers. Splenocytes from antibiotic-treated mice stimulated ex vivo exhibited increased production of TNF. Epidemiological analysis revealed a positive correlation between global antibiotic consumption and IBD incidence.

**Discussion:**

Our findings demonstrate that antibiotic-mediated dysbiosis results in significantly altered short-chain fatty acid levels but immune homeostasis in spleen and MLNs at steady state is mostly preserved. Non-specific activation of splenocytes ex vivo, however, revealed mice with perturbed microbiota had significantly elevated production of TNF. Thus, this highlights antibiotic-mediated disruption of the gut microbiota may program the host towards dysregulated immune responses, predisposing to the development of TNF-associated autoimmune or chronic inflammatory disease.

## Graphical Abstract

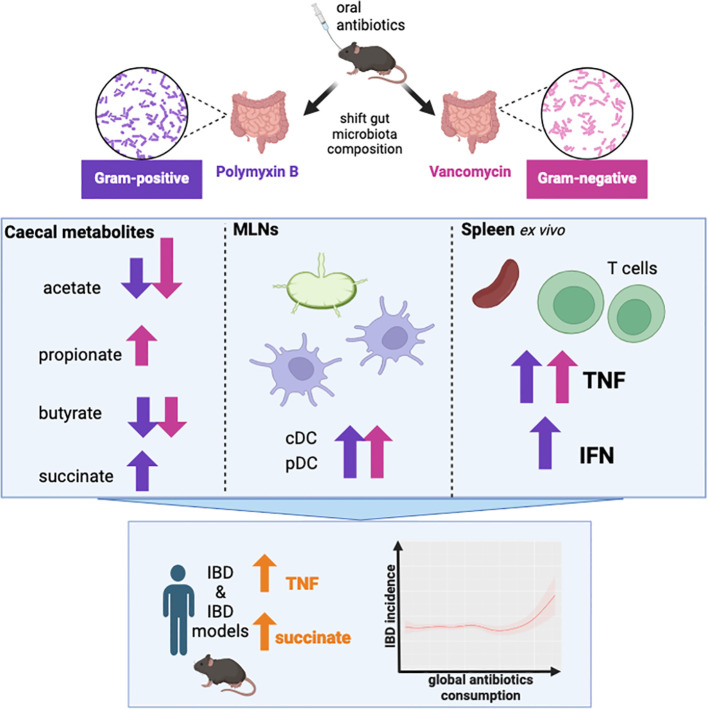

## 1 Introduction

The gut microbiota comprises trillions of microorganisms, predominantly bacteria, which have a profound impact on host physiology, including on metabolism and immunity. Studies of germ-free mice reveal that the absence of gut microbiota results in impaired development across multiple bodily systems, particularly the immune system (1). Gut bacteria are essential for immune cell development, including regulatory T cells (Tregs) (2) and T helper 17 cells (3), and they play a key role in regulating the function of both innate (4, 5) and adaptive immune cells (6).

Although there is no consensus as to what constitutes a healthy or ideal microbiota composition, dysbiosis - defined as detrimental changes to the gut microbiota - has been implicated in a range of diseases including allergies and autoimmune diseases (7). Most notably, dysbiosis is strongly associated with the rise of chronic diseases, such as metabolic syndrome, cardiovascular disease and obesity (8). A growing body of research suggests that dysbiosis not only contributes to the onset and progression of disease (9) but may also influence effectiveness of treatments (10).

The gut microbiota can affect host immunity through the release of metabolites, which interact with specific receptors expressed on host cells. For example, the fermentation of dietary fibre by gut bacteria generates short-chain fatty acids (SCFAs), which bind to G-protein coupled receptors to modulate immune cells (11). Expression of these receptors, along with other metabolite receptors such as the aryl hydrocarbon receptor and the farnesoid X receptor, are not confined to the gut, but are broadly expressed in distal organs such as the liver, lung and brain (12, 13). This widespread distribution accounts in part for the systemic impact of the gut microbiota on the host.

Bacteria can be classified as gram-negative (G-) and gram-positive (G+), based on their membrane structure, and the host immune system has evolved to effectively discriminate between these distinct bacterial components. For example, lipopolysaccharide (LPS) from G- bacteria can activate Toll-like receptor 4, while peptidoglycans and polysaccharides, predominantly from G+ bacteria, activate receptors such as NOD-like receptors and Toll-like receptor 2 (14, 15). Although the immune response to G- or G+ bacteria has been extensively studied in the context of infections (16, 17), the effects of a skewed commensal composition are less well understood. Emerging research indicates that dysbiotic shifts towards predominantly G+ or G- bacteria are associated with distinct diseases (18–21), highlighting the need for further investigation into how gut bacterial composition influences health and disease.

Antibiotics can be a useful tool in the study of gut-microbiota host interactions, allowing researchers to examine the effects of specific bacterial communities on the host. Alongside this, global antibiotic use is rising and raises significant concerns about their impact on gut microbiota. Antibiotics can significantly alter microbiota composition, indiscriminately targeting pathogenic or non-pathogenic species, leading to a reduction in diversity and the loss of key species that are beneficial to host health (22). This disruption often results in dysbiosis, and antibiotic exposure has been correlated with chronic disease development (9, 23).

Here we used aimed to use antibiotics as a tool to shift gut microbiota composition to a predominantly G+ or G- composition to interrogate the impacts of a perturbed microbiota on immune homeostasis in the host.

## 2 Methods

### 2.1 Mice and housing

6-week old female C57BL/6 mice (Australian BioResources, NSW, Australia) were housed under specific-pathogen-free conditions in the animal facility of Charles Perkins Centre, The University of Sydney. Mice were given *ad libitum* access to standardised rodent diet AIN-93G (Specialty Feeds, Glen Forrest, Australia) and drinking water. Mice were weighed twice weekly and monitored for humane endpoints. Humane endpoints for this study included greater than 15% loss of bodyweight, as well as observed changes in behaviour such as hunching, ruffled fur, and inactivity persisting over 24 hours. Loose stool consistency over a 24-hour period was also used as a parameter to assess animal wellbeing. All experiments were performed with approval from the University of Sydney Animal Ethics Committee (2023/2308).

### 2.2 Antibiotics administration

To deplete gram-positive or gram-negative gut bacteria, mice were gavaged with either Vancomycin (5mg/mL; targeting gram-positives) or Polymyxin B (7.5mg/mL; targeting gram-negatives) dissolved in PBS, based on previous studies (24, 25) (n=10 mice per group). Mice were also gavaged with the anti-fungal Amphotericin B (1mg/mL in PBS) (Sigma-Aldrich) for 1 week prior to, and throughout the antibiotic regimen. Mice were gavaged twice daily for all antibiotic treatments, in a maximum volume of 200uL per gavage. Control mice (PBS group) were administered PBS for all gavages.

### 2.3 Metabolites quantification

Caecal content was weighed and homogenised in deuterium oxide (Sigma-Aldrich) at a concentration of 100mg/mL. The sample was centrifuged 5 mins at 14 000 xg at 4°C, caecal pellet was retained for DNA extraction. The supernatant was filtered through 3kDa centrifugal filtration unit (Merck Millipore) and added to chloroform-D/methanol-D (Sigma Aldrich). The upper aqueous phase was diluted into sodium triphosphate buffer with 4,4-dimethyl-4-silapentane-1-sulfomnic acid spiked in as an internal standard. Samples were run on a Bruker 600 MHz spectrometer and data was analysed with Chenomx Profiler software (V10.0).

### 2.4 DNA extraction and 16S rRNA sequencing

DNA from pelleted caecal content was extracted using the FastDNA SPIN Kit for Feces (MP Biomedicals) according to manufacturer’s instructions, and quantified with the Nanodrop for normalisation to caecal weight. The V3–V4 region of the 16S rRNA gene was amplified with Q5^®^ High-Fidelity 2X Master Mix (New England Biolabs) using the Pro341F (5′- CCTACGGGNBGCASCAG -3′) and Pro805R (5′- GACTACNVGGGTATCTAATCC -3′) primers as previously described (26). Following amplification, library cleanup was performed using AMPure XP reagent (Beckman Coulter). Amplicon sequencing was done on the Illumina MiSeq system (2x250 bp PE). Data was analysed using the dada2 package (1.30.0) using R software (4.3.1) to generate Amplicon sequence variants (ASV). ASV were classified according to taxonomy using the Ribosomal Database Project classifier (27). Analysis was performed with the phyloseq (1.46.0), vegan (2.6-4) and microbiome (1.24.0) R packages and differentially abundant taxa were analysed using ALDEx2 (1.34.0). Bacterial load was assessed by absolute 16S copy number per gram of caecal content, as described previously (28). Copy number was determined by qPCR with the 16S rDNA universal primers, UniF: GTGSTGCAYGGYYGTCGTCA and UniR: ACGTCRTCCMCNCCTTCCTC, and compared with a standard curve (10^2^ to 10^8^ copies per uL). Fungal load was determined using primers for the Fungal ITS1 rRNA region (ITS1-30F: GTCCCTGCCCTTTGTACACA, ITS1-217R: TTTCGCTGCGTTCTTCATCG) (29) and the 18S region (F: GGRAAACTCACCAGGTCCAG, R: GSWCTATCCCCAKCACGA) (30), following the relevant cycling conditions.

### 2.5 Immunoglobulin A quantification

Small intestinal content was homogenised at 100mg/mL in PBS with complete Protease Inhibitor cocktail (Roche). Samples were centrifuged 14 000x g for 10 min at 4°C and supernatant collected. Immunoglobulin A (IgA) was assessed with the Mouse IgA Quantitation Set (Bethyl Laboratories) following the manufacturer’s instructions.

### 2.6 Flow cytometry tissue processing

Mice were euthanised via CO_2_ and mesenteric lymph nodes (MLNs) and spleens were collected and processed into single cell suspension. Tissues were disrupted in FACS buffer (2% FCS, 0.5M EDTA in PBS). Red blood cells were lysed for spleen samples using RBC Lysis Buffer 10X (Biolegend). Cell suspensions were passed through a 100µm mesh filter and resuspended in FACS buffer for counting with trypan blue (0.4%) on a haemocytometer to exclude dead cells.

### 2.7 Flow cytometry staining

Cells were stained for viability using LIVE/DEAD™ Fixable Blue Dead Cell Stain (ThermoFisher Scientific), and non-specific binding was prevented using anti-mouse CD16/32 (BioLegend). Surface antigen and intracellular staining was performed using antibodies listed in **Table 1**. For intracellular staining of cytokines and FoxP3, cells were fixed and permeabilised using the Foxp3/Transcription Factor Staining Buffer Set (eBioscience) according to the manufacturer’s instructions. Cells were washed and then acquired on the Aurora (Cytek) with SpectroFlo software (Cytek) and data was analysed in FlowJo (Treestar Inc. Ashland, OR, USA), based on the gating strategies in **Supplementary Figures 4–9**.

**Table 1:** Flow cytometry antibodies.

Antibody	Manufacturer	Catalog #	Clone
anti-mouse B220 BUV661	BD	612972	RA3-6B2
anti-mouse CD3 PE-CF594	BD	562286	145-2C11
anti-mouse CD4 BV750	BioLegend	100467	GK1.5
anti-mouse CD8 BUV805	BD	612896	53-6.7
anti-mouse CD11b BUV395	BD	563553	M1/70
anti-mouse CD11c BV786	BD	563735	HL3
anti-mouse CD25 PE-Cy7	BioLegend	102016	PC61
anti-mouse CD45 Alexa Fluor 700	BioLegend	103128	30-F11
anti-mouse NK1.1 PE/Cy5	BioLegend	108716	PK136
anti-mouse F4/80 BV711	BioLegend	123147	BM8
anti-mouse FoxP3 APC	Miltenyi	130-111-601	REA788
anti-mouse Ly6C FITC	BD	553104	AL-21
anti-mouse Ly6G BV650	BioLegend	127641	1A8
anti-mouse MHCII BV510	BioLegend	107636	M5/114.15.2
anti-mouse TCRγδ APC-Fire 750	Biolegend	118136	GL3
anti-mouse FoxP3 Vio515	Miltenyi	130-111-603	REA788
anti-mouse IFN-γ BV650	BioLegend	505832	XMG1.2
anti-mouse IL-10 APC	BioLegend	505010	JES5-16E3
anti-mouse IL-17 PE	BioLegend	506904	TC11-18H10.1
anti-mouse TNF-a BV510	BD	563386	MP6-XT22
anti-mouse MHCII Pacific Blue	BioLegend	107620	M5/114.15.2
anti-mouse Ly6C BV605	BioLegend	128036	HK1.4
anti-mouse CD103 Vio515	Miltenyi	130-111-609	REA789
anti-mouse CD11b PerCP	BioLegend	101230	M1/70
anti-mouse IL-6 PE	BioLegend	504504	MP5-20F3
anti-mouse F480 PE/Cy7	BioLegend	123113	BM8
anti-mouse iNOS APC	eBioscience	17-5920-82	CXNFT

### 2.8 *In vitro* stimulations

Splenocytes (10^6^ cells) were stimulated with either lipopolysaccharide (LPS) (100ng/mL) (Invivogen) for 48 hours, or with phorbol 12-myristate 13-acetate (PMA) (50ng/mL) (Sigma Aldrich), ionomycin (500ng/mL) (Sigma Aldrich), and brefeldin A (BFA) (5µg/mL) (Sigma Aldrich) for 4 hours. Cells were incubated at 37°C 5% CO2, then washed and stained for intracellular cytokines as described above.

### 2.9 Data collection and processing

Global antibiotic consumption data was sourced from the Global Research on Antimicrobial Resistance (GRAM) database (31). Antibiotic usage was documented as defined daily doses per 1000 population per day (DDD/1000 inhabitants/day). Age-standardised incidence rates of inflammatory bowel disease (IBD) was collated from Global Burden of Disease (GBD) Study 2021 (https://vizhub.healthdata.org/gbd-results/). Global gross domestic product (GDP) data was obtained from the Maddison project (32). Countries or timepoints without available data were excluded from analysis, and the final dataset included data from close to 200 countries, spanning the years 2000 to 2018.

### 2.10 Generalised additive models

Details of analysis were adapted from our previous works (33, 34). Generalised additive model (GAM) is a statistical tool facilitating the simultaneous analyses of multiple parameters, including their plausible interactions and non-linear effects (35–37). They are modelled using non-parametric smoothed functions, which are generally in forms of splines and represent flexible manners to examine non-linear associations. In this study, GAMs were used to model age-standardised IBD incidence rates, using antibiotic consumption, GDP and time as predictors with the “*gam*” function from the *mgcv* package (37, 38). When modelling, countries that the data were based on were corrected as random effects. An array of GAMs were run using aforementioned predictors as well as their different combinations, capturing their individual, additive and interactive effects. Effects from each parameters were modelled as smooth terms with the “s()” function, and their interactions were modelled with the “ti()” or “te()” functions, considering their different scales, such as antibiotic consumption and GDP. Modelling results were evaluated following the Akaike information criterion (AIC) (39), and the one with the lowest AIC was chosen.

### 2.11 Statistics

Comparisons between groups were performed with GraphPad Prism (v10) with ordinary one ANOVA, repeated measures two-way ANOVA, or a mixed-effects model with Geisser-Greenhouse correction, followed by Tukey’s multiple comparisons test. Differences were considered statistically significant when p<0.05, with *p<0.05, **p<0.01, ***p<0.001, **** p<.0001

## 3 Results

### 3.1 Both polymyxin B and vancomycin treatments significantly alter gut microbiota composition and diversity

**Figure 1:**
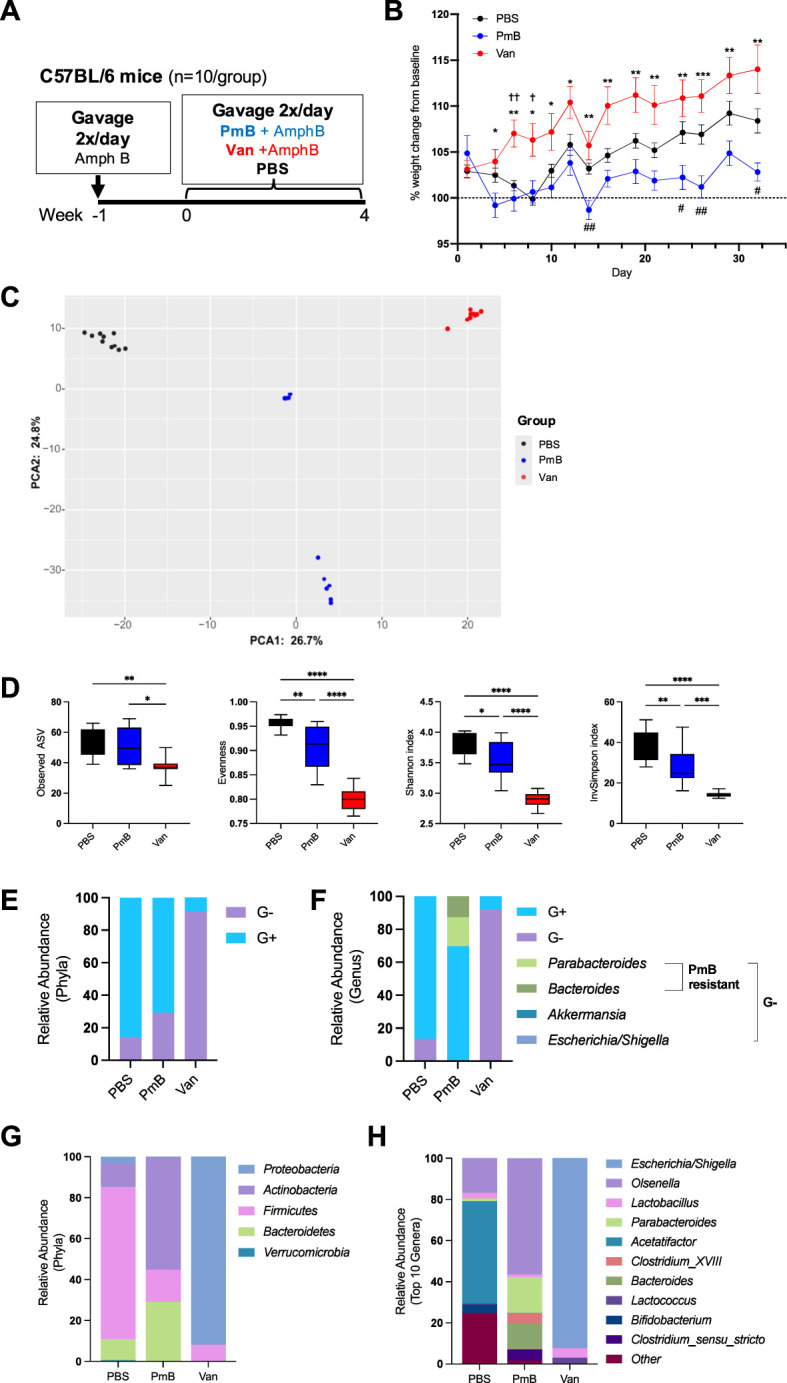
aqr rid="aq1" boffset="10"/>Administration of Polymyxin B or vancomycin significantly alters microbiota composition, eliminating to a G+ or G- taxa. **(A)** Diagram of experimental set up. C57BL/6 mice were gavaged amphotericin B (Amph B) (1mg/mL) 1 week prior to commencing 2x daily gavages of vancomycin (Van) (5mg/mL), Polymyxin B (PmB) (7.5mg/mL), or PBS, for 4 weeks (n=10 mice per group). **(B)** Bodyweight as percent of baseline weight, using mixed-effects model with Geisser-Greenhouse correction, followed by Tukey’s multiple comparisons test. * indicates differences between Van and PmB, † between Van and PBS, # between PmB and PBS **(C)** Differences in microbiota composition shown as principal component analysis (PCA) of Aitchison distance. **(D)** Alpha diversity measures of microbiota composition, showing richness (observed ASV), Evenness, Shannon Diversity index and Inverse Simpson index. **(E)** Phyla and genera were classified as gram-positive (G+) or gram-negative (G-) and relative abundance was graphed for phyla **(E)** and genera **(F)**, also indicating PmB-resistant and susceptible strains. **(G)** Relative abundance of bacteria at phyla level **(H)** and genus level (top 10 genera). Data are represented as mean ± SEM. *p<0.05, **<0.01, ***0.001, ****<0.0001, , †<0.05, ††<0.01, #<0.05, ##<0.01, by ordinary one-way ANOVA followed by Tukey’s multiple comparisons test.

In order to understand the broad impact of G- or G+ bacteria on immune homeostasis we used antibiotics to selectively eliminate G+ or G- taxa within the gut microbiota towards a dominantly G+ or G- composition. We utilised Polymyxin B (PmB), a cationic peptide that binds to LPS to weaken the bacterial outer membrane to target G- species and skew the microbiota towards a G+ composition (40). To shift the microbiota towards a G- composition we used vancomycin, which is active against most enteric G+ species. Before beginning the antibiotic regimen, mice were administered the antifungal amphotericin B (1mg/mL) for 1 week, to avoid expansion of enteric fungi that often occurs with antibiotic treatment. Mice were then maintained on amphotericin B and gavaged either vancomycin (5mg/mL), PmB (7.5mg/mL) or PBS twice daily for 4 weeks (**Figure 1A**), to generate microbiota that was G- or G+ dominant, respectively. All three drugs are reported to have poor bioavailability after oral administration, with little systemic absorption (41–47). Therefore, we expect minimal absorption into peripheral circulation, and the drugs’ primary activity to be confined to the gastrointestinal tract. We selected to administer antibiotic treatments via oral gavage to allow for a standardised dose to be delivered and avoid dehydration and severe weight loss often observed if antibiotics are given in drinking water (25, 48). We observed, however, that vancomycin-treated mice had significant increase in percentage weight gain compared to PmB treated mice, and PmB mice tended to have reduced weight compared to PBS mice towards the end of the experiment (**Figure 1B**). Vancomycin has been linked to weight gain in humans (49) and was found to promote food intake and weight gain in mice (50), which may explain this discrepancy. Caecal weight was also significantly increased by vancomycin treatment (**Supplementary Figure 1A**), as previously described (50, 51), likely due to elimination of fibre-digesting bacteria. While histopathological analysis was beyond the scope of this study, previous studies suggest vancomycin does not induce gross changes to the colonic epithelium (52). However, short-term oral vancomycin was found to disrupt the colonic mucus barrier by inducing ER stress in goblet cells that impaired mucus secretion in goblet cells (53), and both vancomycin and PmB may impair colonic permeability (54).

To confirm our treatments were successful in manipulating gut microbiota composition, we extracted caecal DNA and performed 16S rRNA sequencing. Principal component analysis based on Aitchison distance revealed that all three groups had a distinct microbiota composition (**Figure 1C**). Measures of alpha diversity, which evaluate the species richness and evenness within the gut microbiota, were also examined (**Figure 1D**). Mice treated with vancomycin had a significantly reduced richness compared to PBS and PmB-treated mice, as shown by the decreased number of unique amplicon sequence variants (ASV). Observed ASV was unchanged in PmB-treated mice compared to PBS, indicating this treatment did not significantly reduce the number of unique taxa. Other measures of alpha diversity, including Evenness and Shannon and Inverse Simpson indices, were also significantly reduced in antibiotics-treated groups compared to PBS (**Figure 1D**), however, vancomycin-treated mice exhibited a more pronounced reduction across these measures and were significantly lower compared to PmB-treated mice (**Figure 1D**).

Next, to confirm the efficacy of antibiotic treatment, we examined the relative abundance of phyla in the gut microbiota and classified these as G- or G+ (**Figure 1E**). Basally, the composition of PBS microbiota was mostly G+. As expected, vancomycin treatment led to predominantly G- phyla and PmB resulted in predominantly G+ phyla. Surprisingly, PmB-treated mice had a slightly reduced abundance of G+ phyla compared to PBS. Examination of the G- phyla at the genus level for PmB mice revealed that G- species comprised *Parabacteroides* and *Bacteroides* spp (**Figure 1F**). PmB resistance in commensal *Bacteroides* and *Parabacteroides* has been reported previously, and has been attributed to enzyme modifications to the lipid A component of LPS, which disrupts PmB binding (55–57). Remaining G- genera, *Akkermansia* and *Escherichia/Shigella*, were present at relative abundance of <1% (**Supplementary Figures 1B, C**), indicating that susceptible G- species were successfully eliminated by PmB treatment. Altogether, this demonstrates that our treatments were successful in eliminating the majority of susceptible G+ or G- species within the gut microbiota.

To gain further insight into the gut microbiota composition, we examined the relative abundance of Phyla (**Figure 1G**) and Genus (top 10 genera) (**Figure 1H**). The mostly G+ composition of PBS microbiota was attributed to the presence of *Firmicutes*, primarily represented by the genus *Acetatifactor* (**Figures 1G, H**), which is strongly associated with the rodent gut (58, 59). As described earlier, PmB led to expansion of G- phyla *Bacteroidetes* (comprising genera *Bacteroides* and *Parabacteroides*) and G+ *Actinobacteria* (comprising the genus *Olsenella*). Vancomycin resulted in a microbiota primarily composed of G- *Proteobacteria* phyla, which was due to expansion of the genus *Escherichia/Shigella*, which likely represents the proliferation of a commensal or facultatively pathogenic species (pathobiont) within the existing gut microbiota. Altogether these data further confirm that our treatments effectively targeted susceptible bacteria to significantly perturb gut microbiota composition and induce a shift to a G+ or G- dominant composition.

We also measured 16S copy number, as a readout of bacterial load in the gut, which is an influential factor on host immunity (**Supplementary Figure 1D**). We found antibiotics decreased bacterial load compared to PBS mice, and PmB treated mice had significantly reduced load compared to vancomycin treated mice. Thus, the selective antibiotic treatments effectively targeted their respective species and reduced overall bacterial burden in the gut. We also assessed fungal load within the microbiota, which, despite comprising only 1% of gut microbiota, play a critical role in systemic immune development and responses (60, 61). We observed that vancomycin-treated mice had increased fungal load compared to PBS, as quantified by the relative expression of 18S rRNA, however, there were no significant differences between groups for ITS rRNA expression (**Supplementary Figure 1E**). Altogether, these findings reveal the differential impact of select antibiotic treatments on the bacterial and fungal compartments of the gut microbiota.

### 3.2 Differential production of G+ or G- dominant microbiota metabolites and impact on host sIgA

**Figure 2:**
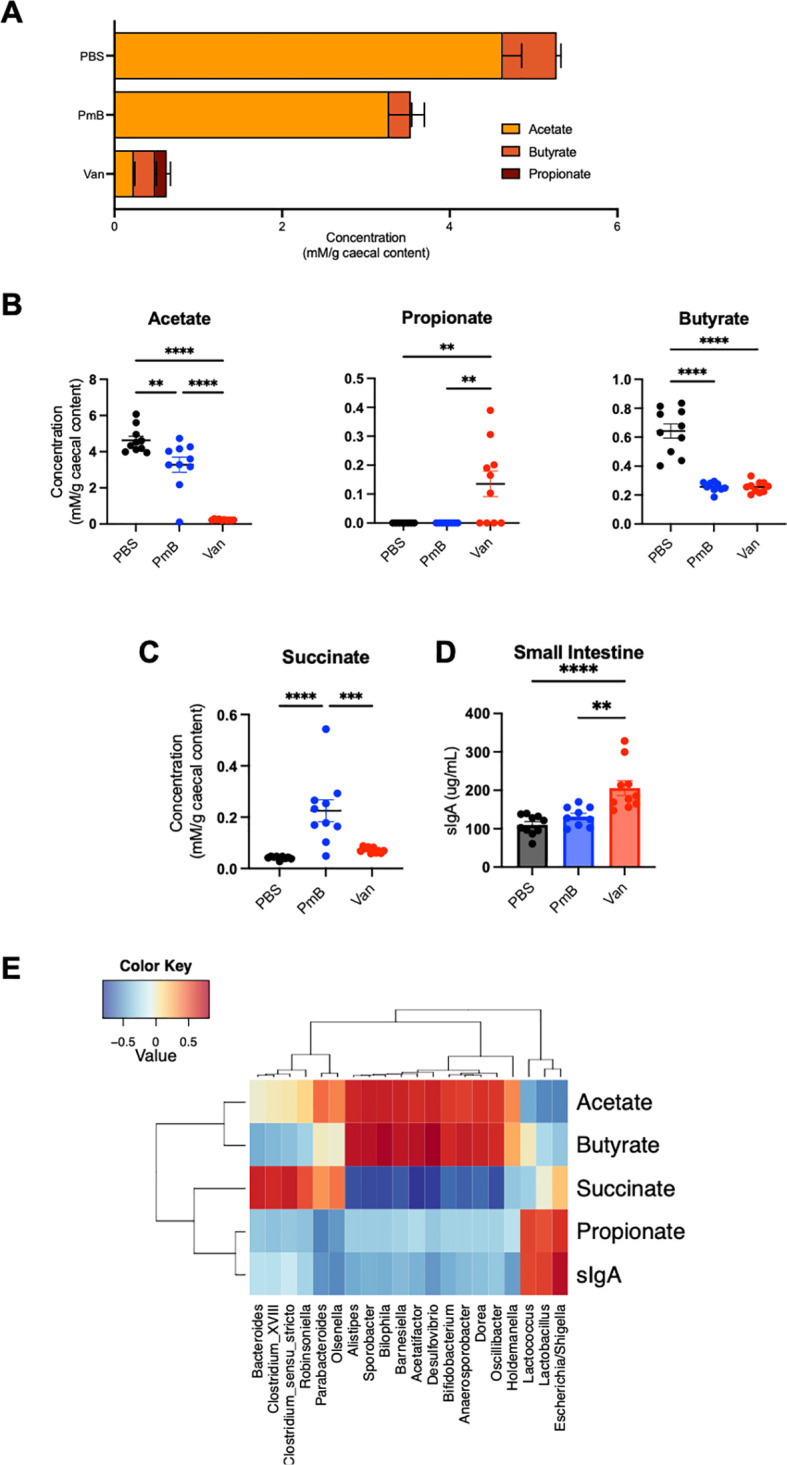
Antibiotic-shifted microbiota have significantly altered metabolite profiles **(A)** Ratios and total amounts of SCFAs (acetate, butyrate and propionate) in caecal content, as quantified by nuclear magnetic resonance (NMR) spectroscopy. **(B, C)** Concentrations of acetate, propionate, butyrate, and succinate in caecal content. **(D)** Levels of sIgA in small intestinal content, as quantified by ELISA. **(E)** Correlation between metabolites, sIgA level, and 16S genera results. Data are represented as mean ± SEM with **p<0.01, ***0.001, ****<0.0001 by ordinary one-way ANOVA followed by Tukey’s multiple comparisons test.

Gut microbiota-derived metabolites, particularly SCFAs, profoundly influence host physiology and immunity. To assess how antibiotic-mediated changes in microbiota composition impact the production of these metabolites, we quantified the levels of caecal SCFAs by nuclear magnetic resonance spectroscopy. We examined levels of the major SCFAs, acetate, propionate, and butyrate, as these play a key role in promoting tolerance (11, 62). Moreover, the majority of SCFA-generating bacteria belong to the predominantly G+ phyla *Firmicutes* (63), which was decreased in abundance in antibiotic-treated groups. Accordingly, these mice had decreased total SCFA levels, with vancomycin-treated mice having the most altered ratios of SCFAs (**Figures 2A, B**). Compared to PBS, acetate was significantly decreased in PmB-treated mice, and close to undetectable in vancomycin-treated mice (**Figure 2B**). Surprisingly, a proportion of vancomycin mice had significantly elevated propionate levels, whereas propionate was undetectable in the PBS and PmB mice (**Figure 2B**). Butyrate was reduced in both antibiotic-treated groups to a similar extent (**Figure 2B**). Finally, succinate, another metabolite that has significant associations with dysbiosis as well as intestinal inflammation and stress (64, 65), was significantly elevated in PmB mice (**Figure 2C**) compared to other groups.

Next, we measured intestinal secretory Immunoglobulin A (sIgA), a key component of the host’s luminal response to the gut microbiota, which has also been linked to high levels of acetate (66). One function of sIgA is binding to luminal bacteria to maintain exclusion of the microbiota from the host epithelium. sIgA was substantially elevated in vancomycin mice, approximately two-fold compared to other groups (**Figure 2D**) suggesting a potential expansion of colitogenic bacteria (67).

Finally, we performed a correlation analysis between bacterial-derived metabolites, host sIgA, and the composition of the gut microbiota (**Figure 2E**). We identified a positive correlation with acetate and butyrate with some G- genera (*Desulfovibrio, Alistipes, Oscillibacter*) and many G+ genera (including *Bifidobacterium*, *Sporobacter, Acetatifactor, Anaerosporobacter*, and *Dorea*). Similarly, succinate levels correlated with both G- taxa (*Bacteroides* and *Parabacteroides*) and G+ phyla *Firmicutes* (including *Clostridia* spp). Although propionate production is commonly correlated with G- *Bacteroides* in human microbiota (68), we observed propionate correlated highly with G- *Escherichia/Shigella* as well as G+ *Lactococcus* and *Lactobacillus* spp, all of which have the capacity to generate propionate (69, 70). Similarly, sIgA correlated strongly with *Escherichia/Shigella*, *Lactococcus* and *Lactobacillus* spp, in line with a previous study in humans treated with vancomycin (71). Together these findings suggest that perturbing microbiota composition can dramatically alter gut microbiota-derived metabolites, some of which may be attributed to the large reduction in *Firmicutes* phyla we observed.

### 3.3 Antibiotic-mediated dysbiosis predominantly affects dendritic cells in the mesenteric lymph nodes

As the mesenteric lymph nodes (MLNs) drain the gut and are the critical sites of antigen-specific response against gut bacteria, we first assessed impact of antibiotic-mediated changes in gut bacteria on this site. To identify how these changes may influence the local immune system in the gut, we quantified proportions and absolute numbers of innate and adaptive immune cells in the MLNs (gating strategy shown in **Supplementary Figure 4**).

**Figure 3:**
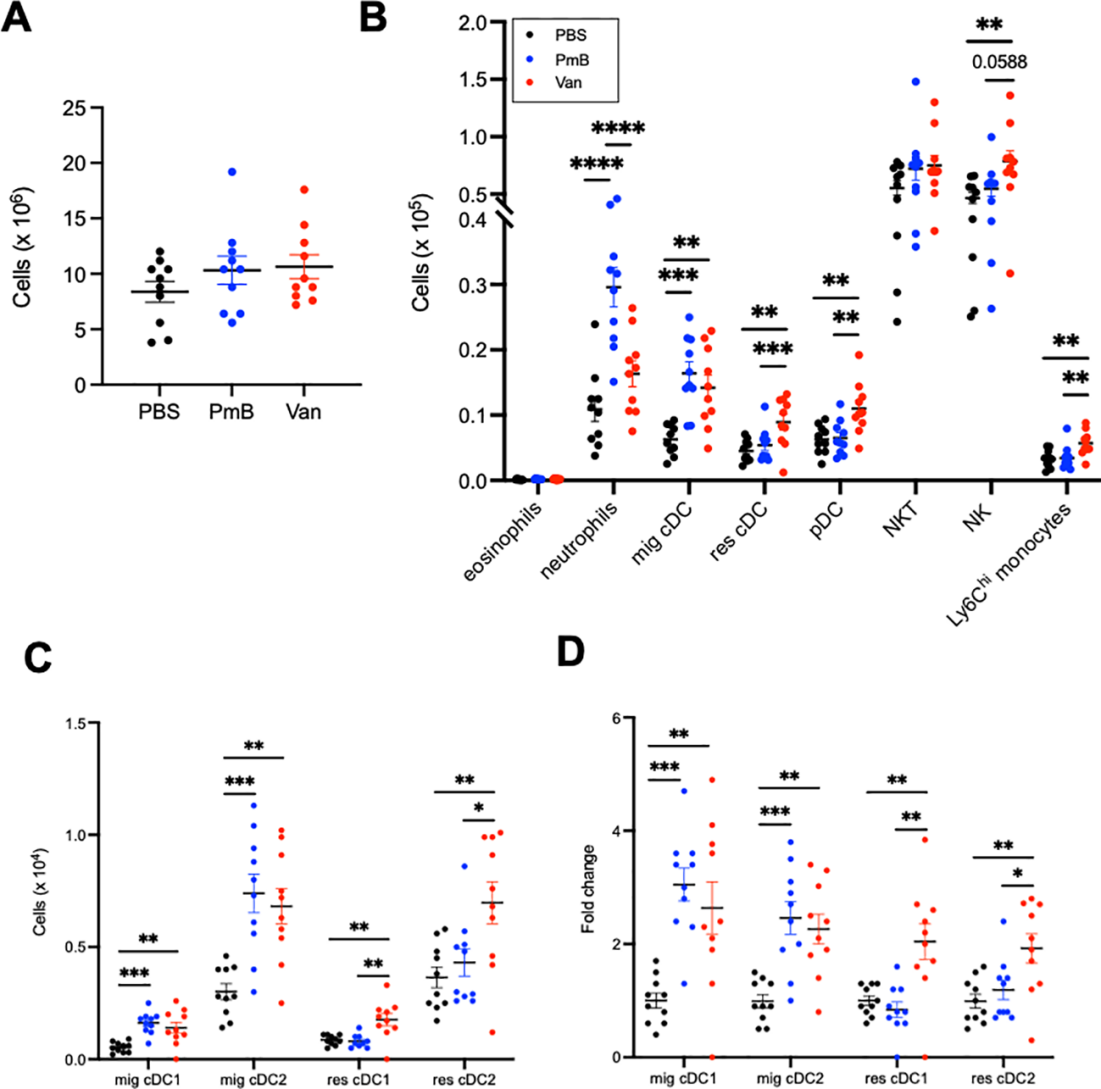
Dendritic cells in mesenteric lymph nodes are mainly impacted by antibiotics-induced dysbiosis **(A)** Total cellularity of the mesenteric lymph nodes (MLNs). **(B)** Absolute cell numbers of eosinophils, neutrophils, migratory conventional dendritic cells (mig cDC), resident cDC (res cDC), plasmacytoid dendritic cells (pDC), Natural Killer T cells (NKT) and Natural Killer cells (NK), and Ly6C^hi^ monocytes were quantified by flow cytometry. **(C)** Numbers of migratory and resident cDC1 (CD11b^-^ CD8^+^) and cDC2 (CD11b^+^CD8^-^) subsets **(D)** and fold change across groups. Data are represented as mean ± SEM with *p<0.05, **<0.01, ***0.001, ****<0.0001 by ordinary one-way ANOVA followed by Tukey’s multiple comparisons test.

Total MLN cellularity was similar among groups (**Figure 3A**), suggesting changes to gut microbiota composition did not induce a localised immune response in this site. We observed that compared to PBS control, a antibiotics-perturbed microbiota impacted the proportions of a range of innate and adaptive immune cell subsets (**Supplementary Figures 2A, B**). Both antibiotics treated groups had increased proportions of migratory conventional DCs (MHCII^hi^ cDCs), and PmB mice had increased neutrophils compared to vancomycin and PBS mice. Vancomycin treatment increased proportions of plasmacytoid DCs (pDCs), resident cDCs, Natural Killer (NK) cells, Ly6C^+^ inflammatory monocytes compared to the other groups, and led to decreases in TCRγδ^+^ T cells compared to PBS and decreased CD4^+^ T cells compared to PmB mice(**Supplementary Figures 2A, B**). For absolute cell number, we found mainly innate cells were influenced by antibiotic treatment (**Figure 3B**, **Supplementary Figure 2C**). PmB treated mice had increased neutrophils compared to PBS and vancomycin mice, while vancomycin mice had increased NK cells and Ly6C^+^ monocytes compared to the other groups (**Figure 3B**). We found both antibiotic-treated groups had significantly increased numbers of migratory DCs compared to PBS, while vancomycin mice had increased numbers of resident cDCs and pDCs compared to PmB and PBS mice (**Figure 3B**). DCs are key antigen-presenting cells that are crucial for the development of antigen-specific responses in the gut and in the induction of tolerance, and cDCs and pDCs differ in their ontogeny as well as their functional role, with cDCs being highly efficient in antigen-presentation and activation of naïve T-cells whereas pDCs play a key role in production of type I interferons and the anti-viral response (72). Altogether our findings suggest that skewing the microbiota towards a dysbiotic composition mainly impacts innate cell numbers, primarily DCs.

Migratory and resident cDCs can be further classified into cDC1 (CD8a^+^CD11b^-^) and cDC2 (CD8a^-^CD11b^+^) subsets, which are also functionally specialised: cDC1 are key in the cytotoxic immune response and presentation to CD8^+^ T cells, and cDC2s are key in helper T cell responses and presentation to CD4^+^ T cells. Both migratory cDC subsets were increased in proportion and number for antibiotics-treated mice (**Figure 3C**, **Supplementary Figure 2D**). For vancomycin mice, both resident cDC1 and cDC2 subsets were also increased in number compared to other groups (**Figure 3C**). The fold-change for increased migratory cDC numbers was approximately 3 times greater for both antibiotics-treated groups compared to the PBS group, while the increase in resident cDC in vancomycin mice was twice that of PBS and PmB groups (**Figure 3D**). Altogether, these data indicate that a dramatically altered microbiota composition impacts innate cells in the gut-draining lymph nodes, and a PmB- or vancomycin-skewed microbiota appear to differentially impact neutrophils and pDCs, but both increasing migratory cDC numbers.

As we observed DC numbers were highly impacted by a skewed microbiota, we next examined the impact on T cells. Neither the proportion nor the number of total T cells, CD4^+^ T cell or CD8^+^ T cell subsets were altered in mice with perturbed microbiota compared to PBS (**Supplementary Figures 2B, C**), suggesting there was no initiation of an antigen-specific immune response, consistent with the unchanged MLN cellularity. This further confirms that antibiotic treatments perturbing the microbiota mainly impact innate, and not adaptive cell subsets, in the MLNs.

Altogether these results suggest that an antibiotics-perturbed microbiota increase recruitment of DC to the MLNs, but without initiation of an antigen-induced T cell response.

### 3.4 Perturbed gut microbiota minimally impacts cell numbers in the spleen but increases their pro-inflammatory profile

To determine if a dramatically skewed microbiota had a systemic impact on immunity, we next investigated proportions and numbers of innate and adaptive cells among splenocytes, as well as their cytokine secretion profile.

**Figure 4:**
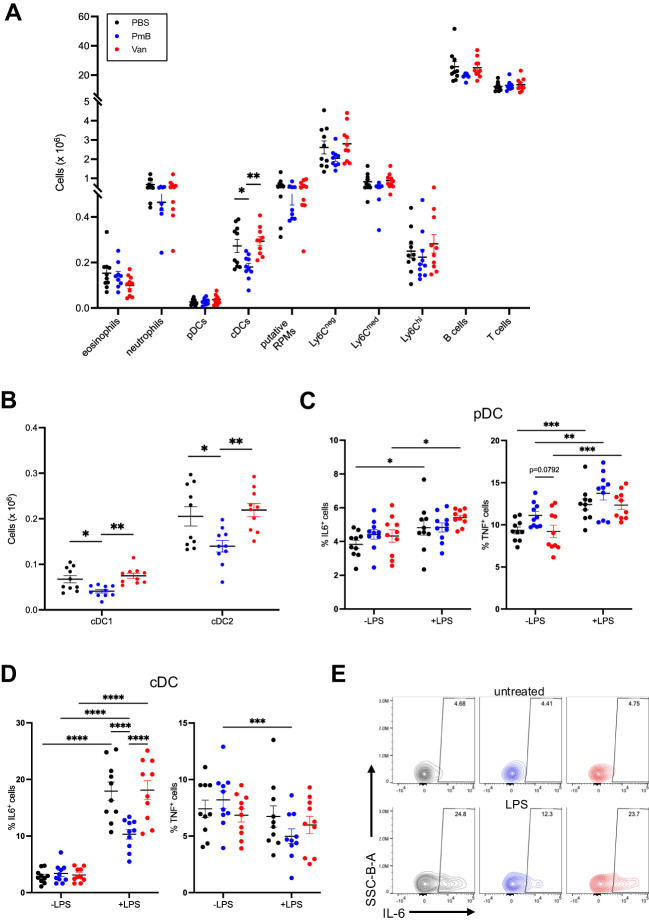
Perturbed microbiota has minimal impact on spleen immune composition **(A)** Absolute cell numbers of innate and adaptive cell subsets in the spleen, including eosinophils, neutrophils, plasmacytoid dendritic cells (pDCs), conventional dendritic cells (cDCs), putative red pulp macrophage (RPMs), monocyte subsets (Ly6C^hi,^Ly6C^mid^ Ly6C^neg^), B cells and T cells as quantified by flow cytometry. **(B)** Numbers of cDC1 (CD11b^-^ CD8^+^) and cDC2 (CD11b^+^CD8^-^) subsets in the spleen. **(C)** Expression of IL-6 and TNF in pDCs stimulated with lipopolysaccharide (LPS) for 48 hours *in vitro*, as quantified by flow cytometry. **(D)** Expression of IL-6 and TNF in cDCs stimulated with LPS for 48 hours *in vitro*, as quantified by flow cytometry and **(E)** representative plots of proportions of IL-6 expressing cells in LPS-stimulated and unstimulated cDCs. Data are represented as mean ± SEM with *p<0.05, **<0.01, ***0.001, ****<0.0001 by ordinary one-way ANOVA followed by Tukey’s multiple comparisons test or repeated measures two-way ANOVA followed by Tukey’s multiple comparisons test.

Similar to the MLN we found no changes in overall splenic cellularity (**Supplementary Figure 3A**). However, mice with perturbed microbiota had altered proportions of immune subsets including eosinophils, cDCs, putative red pulp macrophages (F480^+^Ly6C^-^ cells) and Ly6C^med^ monocytes, as well as T cell subsets (**Supplementary Figures 3B, C**; gating strategy shown in **Supplementary Figure 5**), suggesting the systemic impact of the microbiota shift. As in the MLNs, DC subset numbers were primarily impacted, with a significant reduction of total cDCs as well as cDC1 and cDC2 subsets in PmB-treated mice compared to other groups (**Figures 4A, B**). Contrary to the MLNs, we found no change to pDC numbers.

To further investigate the impact of antibiotic-mediated dysbiosis on immune function, we quantified the cytokine secretion before and after challenge *in vitro*. To do this, we stimulated splenocytes with LPS from *E. coli* for 48 hours and examined the intracellular production of pro-inflammatory cytokines IL-6 and TNF by flow cytometry (**Figures 4C–E**; gating strategy shown in **Supplementary Figure 6**). As expected, LPS treatment in pDC from PBS-treated mice led to increased production of IL-6 but this was to the same extent as pDC from vancomycin mice (**Figure 4C**). Interestingly, there was no effect of LPS on pDCs from PmB mice (**Figure 4C**). This differential effect of the gut microbiota on IL-6 was not observed for TNF, as pDCs across groups produced comparable levels of TNF upon LPS treatment (**Figure 4C**). LPS stimulation of cDCs induced robust IL-6 expression for all groups, however, cDCs from vancomycin mice showed reduced expression compared to PBS and PmB mice (**Figure 4D**). Although we did not measure circulating or faecal LPS levels in our mice, which may reflect differences in LPS tolerance, the fact that LPS is the primary antigen of the G- membrane suggests that reduced LPS levels in PmB mice may affect the DC response to this stimulus. This may be supported by the fact that *Bacteroides* is the only G- phyla present in PmB-treated microbiota, and *Bacteroides*-derived LPS is well-known to be less immunogenic than *E. coli*-derived LPS (73). Although PmB is known for its endotoxin-binding capability, the low likelihood of systemically circulating PmB suggests that the gut microbiota remains a critical source of antigens that shapes immune responses in distal organs. LPS stimulation did not induce TNF expression in cDCs, and there was in fact a slight reduction in TNF levels in PmB mice (**Figure 4D**). Overall, these results indicate that a PmB treated microbiota primarily impacts cDC activation by LPS, possibly reducing cDC capacity to respond to stimuli from immunogenic G- bacteria.

**Figure 5:**
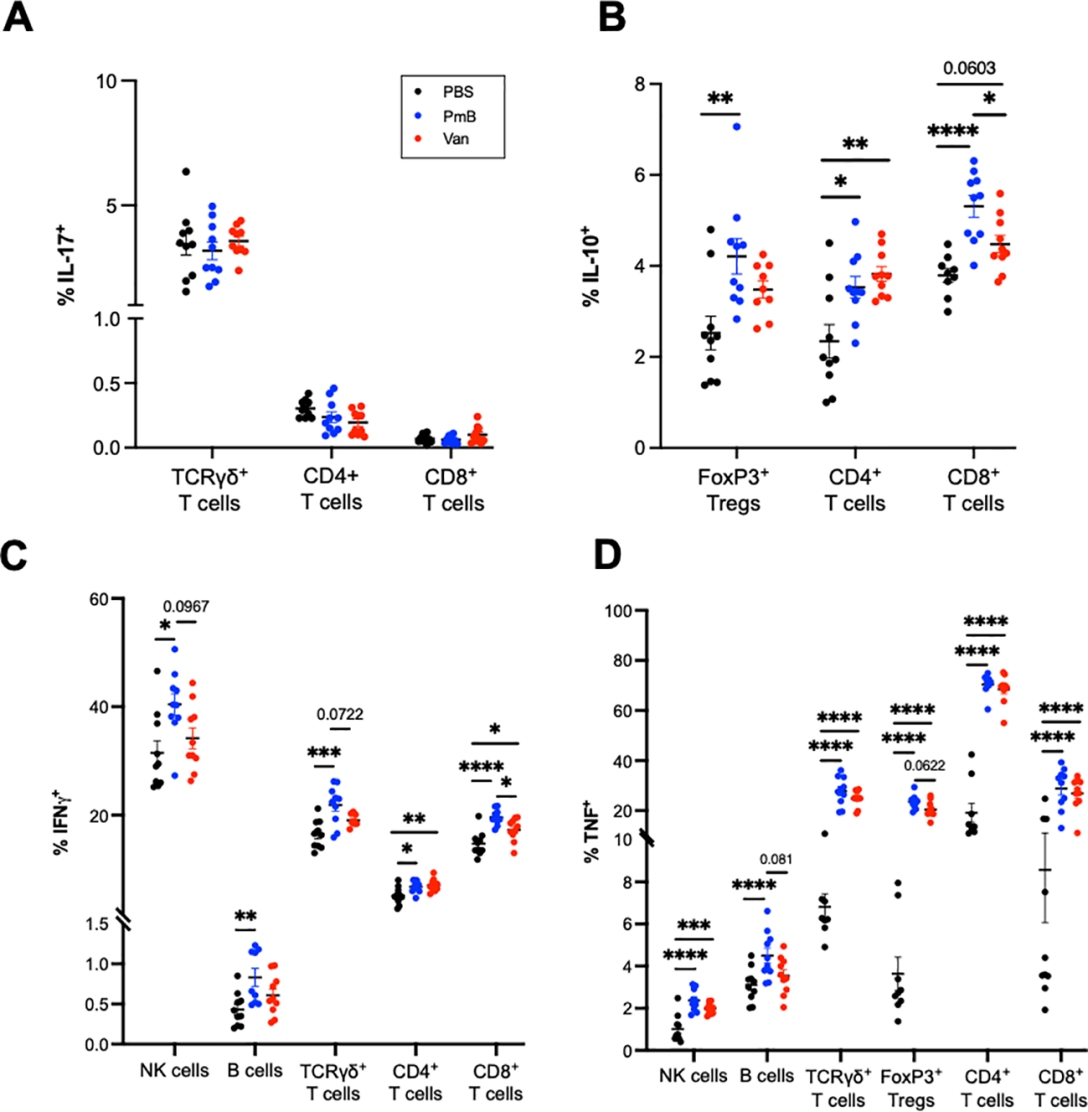
Adaptive splenocytes from antibiotics-shifted microbiota shifted mice show greater cytokine expression under stimulation conditions. Splenocytes were stimulated with PMA/ionomycin/BFA for 4 hours and cytokine expression was quantified by flow cytometry. **(A)** IL-17 expression in TCRγδ^+^, CD4^+^ and CD8^+^ T cells **(B)** IL-10 expression in Tregs, CD4^+^ and CD8^+^ T cells. **(C)** IFN-γ expression in NK cells, B cells, and TCRγδ^+^, CD4^+^ and CD8^+^ T cells **(D)** TNF expression in NK cells, B cells, TCRγδ^+^ T cells, FoxP3^+^ Tregs and CD4^+^ and CD8^+^ T cell subsets. Data are represented as mean ± SEM with *p<0.05, **<0.01, ***0.001, ****<0.0001 by ordinary one-way ANOVA followed by Tukey’s multiple comparisons test.

To further examine the impact of antibiotic-mediated dysbiosis on immune cells, we measured the expression of the cytokines IL-17, IFN-γ IL-10 and TNF, in splenocytes after PMA/ionomycin stimulation (**Figure 5**; gating strategy and FMOs shown in **Supplementary Figures 7-9**). Many studies have identified G+ and G- bacteria induce differing cytokine immune profiles *in vitro* (74–77), as well as during sepsis (78), highlighting the capacity of the immune system to discriminate between the two classes of bacteria. We first examined levels of IL-17 and IL-10, as these cytokines are highly influenced by gut microbiota composition (1, p. 20). The development of IL17-producing cells TCRγδ^+^ T cells in the intestines, as well as in the liver and lungs, is regulated by the gut microbiota (79, p. 201; 3, 80). We observed antibiotics-treated mice showed no changes in IL-17^+^ cells for CD4^+^ T cells. TCRγδ^+^ and CD8^+^ T cells (**Figure 5A**). The induction of intestinal Th17 cells is highly dependent on a subset of G+ bacteria known as segmented filamentous bacteria (3), thus while we did not observe changes in IL-17 production in the spleen, measuring production of IL-17 in gut-local T cells may provide more relevant insights into the influence of antibiotics-perturbed microbiota. The anti-inflammatory cytokine IL-10 is primarily derived from Tregs that are induced by tolerogenic DCs. The generation of tolerogenic DCs is also strongly associated with G+ species, highly prevalent in PmB treated mice (81, 82). In line with this, we found PmB mice had significantly elevated IL-10 expression across T-cell subsets, including Tregs, non-Treg CD4^+^ cells, as well as CD8^+^ T cells (**Figure 5B**). Vancomycin mice also had elevated proportions of non-Treg IL10^+^ CD4^+^ T cells and tended to have elevated IL10^+^ CD8^+^ T cells (p=0.0603) compared to PBS mice (**Figure 5B**).

Finally, we examined the expression of IFN-γ and TNF, pleiotropic cytokines that are instrumental in regulating immune response and also associated with autoimmune diseases. IFN-γ, a type II interferon, is thought to contribute to the pathological intestinal inflammation seen in irritable bowel disease (IBD) (83, 84) and TNF is widely recognised for its involvement across autoimmune conditions such as IBD, arthritis, psoriasis, and systemic lupus erythematosus (85). Previous studies have also identified increases in TNF mRNA in colon tissue after PmB and vancomycin treatment (54, 86). While these studies differed from ours in antibiotic administration methods and resulting microbiota compositional changes, they are suggestive of a link between antibiotics exposure and a pro-inflammatory response. We observed that compared to PBS, PmB mice showed IFN-γ production across a range of subsets including NK cells, TCRγδ ^+^ T cells, CD4^+^ and CD8^+^ T cells, as well as B cells (**Figure 5C**). IFN-γ^+^ cells also tended to be increased compared to vancomycin mice for NK, TCRγδ^+^,and CD8^+^ T cell subsets. Vancomycin mice exhibited IFN-γ CD8^+^ T cells only, compared to PBS (**Figure 5C**). Strikingly, we observed that an antibiotics-skewed microbiota, resulted in a significantly increased TNF expression across a variety of cell types, including NK cells, TCRγδ^+^ T cells, Tregs, CD4^+^ T-cells and CD8^+^ T-cells (**Figure 5D**). This increase was most prominent for TCRγδ^+^ and CD4 subsets, where the average % of TNF positive cells was more than double the PBS group (**Figure 5D**). Altogether these data show that while PmB may promote IL-10 expression, contributing to a protective response, both PmB and vancomycin-treated microbiota significantly upregulate TNF expression, which may potentially exacerbate inflammation.

Given antibiotic-treated mice showed significant upregulation of TNF and IFN-γ, critical mediators of IBD, this prompted us to examine the link between antibiotic consumption and global burden of IBD. Previous studies have identified associations between antibiotic exposure and development of chronic inflammatory diseases, such as metabolic disease, allergies, and autoimmune conditions, including colitis (23). Therefore, we gathered antibiotic usage data from the Global Research on Antimicrobial Resistance database, as well as age-standardised IBD incidence rates from Global Burden of Disease 2021 (GBD2021) and analysed their relationship using a generalised additive model (GAM). GAMs allow for comprehensive analysis of multiple parameters, simultaneously covering their individual, interactive effects, including non-linear effects. Our GAM also adjusted for confounders such as socioeconomics, reflected by gross domestic product (GDP), and corrected for inter-country differences as random effects (see **Supplementary Information** in **Supplementary Data Sheet 1**).

**Figure 6:**
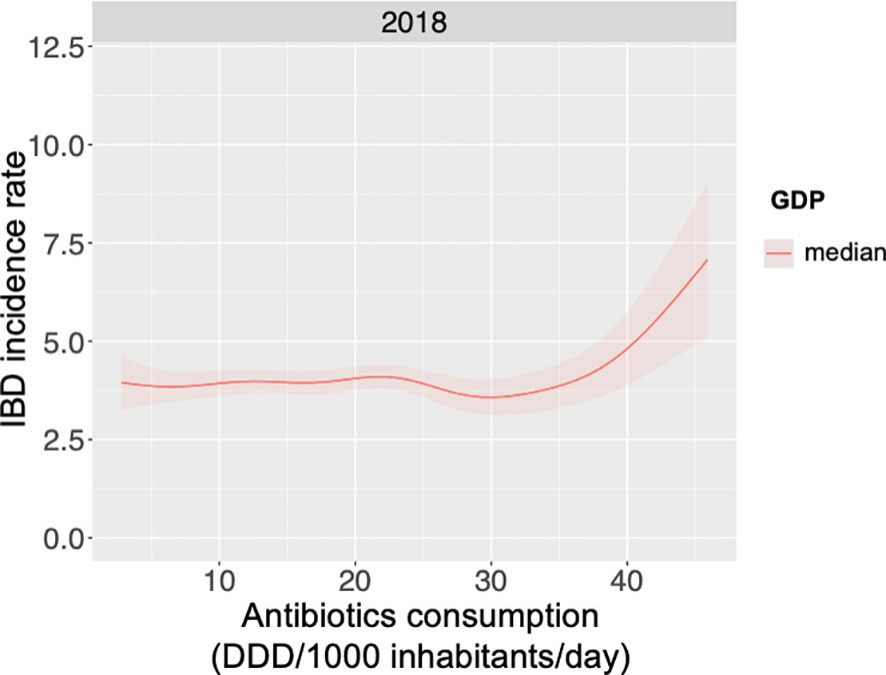
Global IBD incidence increases with increasing antibiotics consumption for median GDP. Global antibiotic consumption data, as Defined Daily Doses per 1000 inhabitants per day (DDD/1000 inhabitants/day) for 2018, was collected along with age-standardised incidence rates of irritable bowel disease (IBD) and data of gross domestic product (GDP). A generalised additive model was used to examine the relationship between antibiotic consumption, GDP and IBD incidence, with results for the year 2018 and median GDP presented.

**Figure 6** presents the modelling results for 2018, the most recent timepoint with complete data coverage, illustrating the predicted response curve of IBD incidence rate as a function of antibiotic consumption (Defined Daily Dose per 1000 inhabitants) at the median-grouped GDP. Globally, increased antibiotic consumption correlated with higher IBD disease burden. However, this relationship was also influenced by GDP, as the association dissipates at a high GDP bracket (**Supplementary Figure 10**). Here, our global analysis aligns with previous observational studies, which have demonstrated an association with antibiotics exposure and IBD incidence rates (87–89).

Overall, these data suggest a perturbed microbiota primes host cells for heightened pro-inflammatory expression under activation conditions. A PmB-treated microbiota in particular may skew the host towards a Th1-biased phenotype, in line with data that found G+ bacteria preferentially induce IFN-γ and TNF in human PBMCs (90).

## 4 Discussion

The aim of this project was to investigate how antibiotic-mediated shifts in gut microbiota composition, impact the gut microbiota-host immune interaction. We utilised two antibiotic regimens to alter gut flora and found that while gut-derived SCFAs were highly impacted, the profile of immune cells in the spleen and MLNs were largely unchanged, suggesting minimal distal immune perturbations under basal conditions. While the MLNs showed minor changes in specific cell populations, it is important to note we did not assess other components of the mucosal immune system (i.e. intestinal lamina propia or epithelia), which have been shown to exhibit localised changes in response to antibiotics, as discussed below. *Ex vivo* stimulation revealed that adaptive cells from antibiotics-treated mice show greater pro-inflammatory cytokine expression, suggesting that immune function is impacted under challenged conditions. Altogether this highlights the need to better understand how dysbiosis may predispose the host to disease development, and how antibiotics may set the scene for inflammatory disease (**Graphical Abstract**).

While our study focused primarily of the impact on the spleen and MLNs, there is extensive body of literature highlighting their impact on mucosal immunity. For example, vancomycin specifically depletes intestinal Th17 and Treg populations (81, 91), demonstrating the importance of gram-positive segmented filamentous bacteria and *Clostridia* species in the induction of these T cell populations in the intestines. Antibiotics also reduce intestinal epithelial cell proliferation, and deplete innate cell populations, such as macrophages and monocytes in the ileum and colon (4). Notably, these perturbations can remain despite attempts to restore the microbiota via faecal transplantation, revealing their long-term impacts on immune homeostasis. Recent studies also reveal the short-term impact of antibiotics, with even a brief (3-day) course of broad-spectrum antibiotics significantly altering colonic innate lymphoid cell populations (92). As reviewed in Kennedy et al. (1) antibiotics also alter various components of intestinal immunity (such as antimicrobial peptides production, TLR expression, etc.), and the specific alteration varies depending on the antibiotic regimen, tissue examined, and the experimental model (1). For an overview of broad-spectrum antibiotic regimens and their impact on immunity, refer to Kennedy et al. (1),

Antibiotic treatment also impacts systemic immunity beyond the mucosal compartment. For example, disruption of commensal signalling with broad spectrum antibiotics influences haematopoiesis via STAT1 to reduce bone marrow progenitors (93), and also influences basophil development and IgE levels, via MyD88-dependent pathways (94). Scott et al. (95) further showed that antibiotic treatment followed by recolonisation results in persistent changes to the colonic lamina propria, including elevated Th1 responses driven by infiltrating pro-inflammatory macrophages. These macrophages produced high levels of TNF and IL6, contributing to a sustained inflammatory environment and enhancing the Th1 response. Altogether, these findings reveal the widespread and persistent impact of antibiotics, which can significantly perturb multiple immune compartments.

While previous studies have explored the effects of vancomycin and PmB on gut microbiota and host tissues, these studies focused on changes in faecal metabolites (51, 54) and characteristics of colon tissue (86). Methodological differences in these studies also limit the relevance of their findings to the influence of gut microbiota on host immunity. Zhang et al. (51) and Sun et al. (54) found PmB did not alter microbiota composition to be significantly different than vehicle controls, which may have resulted from inconsistent dosing associated with antibiotics in drinking water. Consequently, these studies do not provide clear links between how gut microbiota composition may influence observed changes in metabolites. These studies also used mice younger than 6-weeks old, which may limit relevance to mature adult immune systems. Ran et al. (86) found PmB and vancomycin increased colonic permeability *ex vivo*, and increased TNF expression in colonic tissue. However, their study used a short 7-day regimen of antibiotics in drinking water, and only reported minor changes to PmB microbiota at the genus level. Thus we have aimed to address a critical gap by characterising the long-term (4+ week) impact of antibiotics and how this may influence immune responses in gut local and systemic organs in otherwise healthy mice.

It is also important to note each antimicrobial in our study has been shown to exert immunomodulatory effects on cytokine production *in vitro*. PmB is a protein kinase C inhibitor, and thus would likely decrease cytokine production when directly exposed to T cells (96). Vancomycin appears to have context-dependent effects, with differential regulation of TNF mRNA in primary PBMCs versus the Thp-1 monocyte cell line (97, 98). More recently, vancomycin was found also to directly interact with specific HLA alleles (99) and affect macrophage mitochondrial function (100). Similarly, amphotericin B, while primarily targeting ergosterol in fungal cells, has weak affinity for mammalian cholesterol and TLRs (46, 101). The drug also induces pro inflammatory cytokines such as TNF via the MyD88 pathway, although this may vary according to cell type and drug formulation (101).

Although these drugs demonstrate immunomodulatory effects *in vitro*, the oral administration route in our study makes it unlikely the drugs reached significant circulating concentrations to directly influence distal immune cells. Thus, we attribute the increase of TNF production in splenocytes to perturbations in microbiota-derived signalling, rather than a direct impact of the drugs themselves.

Another important methodological consideration of our study was the lack of an Amphotericin-only control group, to account for potential effects of the mycobiome. Interestingly, we observed vancomycin treatment resulted in increased fungal DNA levels compared to PBS groups, as determined by fungal 18S rRNA expression. Previously, vancomycin impaired gut antifungal immunity Th17 cells in the small intestinal lamina propria, likely due to the loss of segmented filamentous bacteria (102). Although we did not assess intestinal T cells, this impact of vancomycin could explain our observed increase in fungal DNA. Amphotericin B has broad-spectrum antifungal activity, and is commonly used in microbiota depletion studies to prevent fungal overgrowth, rather than achieve complete depletion (103, 104). Hence some alteration in fungal load is not unexpected. However, we cannot fully exclude that changes in mycobiome composition may have occurred due to our treatment, which could be potential confounding factor in our immunological findings.

We observed a significant increase in TNF expression in immune cells from antibiotic-treated mice after non-specific activation, particularly in TCRγδ and CD4^+^ T cells. Although TNF is a key mediator of the host defence response, it also plays a pathological role in autoimmune diseases, with elevated TNF levels observed in arthritis, psoriasis and IBD (83, 105–107). While the exact mechanisms by which TNF may initiate or sustain immune dysregulation in these conditions is still under investigation, TNF can be regarded as the “apex of a pro-inflammatory cascade” (85). This is demonstrated by the effectiveness of anti-TNF therapies which are widely used to neutralise TNF signalling, alleviating symptoms and improving outcomes in chronic inflammatory diseases. Dysbiosis is also considered a component of chronic inflammatory diseases, with the gut microbiota thought to contribute to inflammation and disease progression (108, 109). Previously we found that Imiquimod-induced psoriasis triggered dysbiosis, leading to a microbiota that produced elevated caecal succinate (65). This rise in succinate promoted the proliferation of a homeostatic population of colonic macrophages, which aggravated the symptoms of DSS-induced colitis. Indeed, psoriasis has a significant association with IBD, with evidence suggesting that IBD may be a causal risk factor for psoriasis, though not vice versa (110, 111). This highlights the possibility that alterations to gut microbiota homeostasis could be a fundamental contributor in the development of extra-intestinal autoinflammatory diseases.

Numerous studies have linked antibiotic exposure in high income countries with later development of autoimmune or chronic diseases, particularly when exposure occurs in early life (23). Our findings suggest that an antibiotics-perturbed microbiota results in an immune system that is basally predisposed for a heightened TNF response, which could help explain this association. This could be further explored using an autoimmune disease model, particularly one that may be T-cell mediated such as experimental autoimmune encephalitis or collagen-induced arthritis models (112, 113). While the precise relationship between TNF, gut dysbiosis and autoimmune disease is under investigation, our study highlights this as a crucial area for future research.

The broader context in which antibiotics are used is also key when considering their secondary impacts. For example, administration of the broad-spectrum antibiotic azithromycin (given biannually over two years) was found to reduce all-cause mortality in children under five across several sub-Saharan African countries (114). Thus, while antibiotics exposure may be a contributing factor to chronic disease in low-infection risk environments (such as high GDP countries), they provide significant health benefits in high-infection risk settings. Moreover, the increased basal immune activation we observed in our study may also be beneficial in such high-risk settings, although further investigation is warranted.

Although our findings indicate the immune system may be primed for heightened response, we found that dramatic changes in microbiota composition did not broadly modify host immunity in distal lymphoid organs during homeostasis, except for alterations in DC numbers in the spleen and MLN. Given the rising and widespread use of antibiotics and concerns about their adverse impact on gut flora, this finding is reassuring as it suggests the systemic immune compartment largely maintains homeostasis, despite substantial shifts in the gut microbiota. While we did not examine the recovery of microbiota after the cessation of antibiotics, most healthy humans recover baseline composition and richness two months after antibiotics exposure, but with a significant increase in the prevalence of antibiotic resistance genes (115, 116). Some individuals, however, do not fully recover, maintaining a “scarred” composition that resembles critically ill ICU patients (115). This emphasises the importance of prudent antibiotic usage as well as the need for greater understanding of the long-term consequences of a “scarred” microbiota: whether specific bacteria or metabolites that are important for host health are most likely to be impacted. While we focused on the impact of the gut microbiota on immunity under homeostatic conditions, many have observed that antibiotic-induced disruption to gut flora is associated with worse outcomes in various conditions such as infection, stroke, cancer and allergy (117–121). Therefore, it would be important to investigate our model under conditions particularly where the gut immune system is challenged, such as enteric infection or food allergy, to fully understand the impact of antibiotic-mediated dysbiosis on the host.

We observed primarily DC subsets were altered in the spleen and MLNs. One potential mechanism for these changes could be through microbiota-derived signalling on haematopoiesis. The microbiota drives haematopoiesis primarily via TLR signalling (5, 93, 122) and is necessary for proper neutrophil and NK cell function (123, 124). While some studies suggest antibiotics do not impact DC progenitors in bone marrow (125, 126), others have identified an impact via FLT3L, a key DC growth factor (117). Recently, gut microbiota-derived propionate, delivered via breastmilk, was identified as essential for FLT3L signalling and thus pDC development in the neonatal gut (127). While we did not assess FLT3L expression, this may explain the increase in MLN pDCs we observed only in vancomycin mice who had elevated propionate levels. However, a more likely explanation for our observation is increased DC maturation and migration from the intestinal lamina propria, in response to our antibiotic regimen, which likely induced local inflammation.

We found perturbing the microbiota increased the numbers of migratory cDCs in the MLNs, identified as CD11c^+^ MHCII ^hi^ cells. This finding aligns with previous observations by Diehl et al. (128), where antibiotic-induced dysbiosis during *Salmonella* infection promoted antigen uptake by CD11c^+^ CX_3_CR1^hi^ mononuclear phagocytes, that trafficked to the MLNs to induce a *Salmonella*-specific T cell response (128). Although we did not measure CX_3_CR1 or CD103 (a migratory DC marker) expression, these markers would confirm migratory cDC identity. We did not observe changes to adaptive cell numbers in the MLNs and spleen, suggestive of no overt immune response. However, a comprehensive profile of the mucosal T cell compartment, particularly using approaches to track T cell migration (such as FTY720 to block egress), would be needed to fully assess potential antigen-specific responses to long-term antibiotic-mediated dysbiosis. Notably, the gut microbiota appears to influence vaccine efficacy (129). Human trials examining impact of antibiotics on vaccine efficacy show mixed results but are often limited by small sample size (n<100) (129). However, there is extensive evidence in mice indicating that antibiotics exposure or impaired TLR recognition has a detrimental effect on antibody response (129–131). While the mechanisms underlying this are still under investigation, the gut microbiota seems to have an adjuvant effect: as a source of pattern-recognition receptor ligands for antigen-presenting cells; through production of immunomodulatory metabolites that regulate host cells; and as a source of cross-reactive epitopes (132). Infancy also seems to be the crucial period in which the gut microbiota influences vaccination outcomes, for mice and humans (129), again highlighting the importance of the early life period on life-long immunity and the need for cautious use of antibiotics during this time.

In the study of microbiota-host interactions, SCFA represent a particularly important class of metabolites, given their extensive role in maintaining immune homeostasis and gut health (11). Here, perturbing the microbiota composition reduced overall levels of SCFAs, particularly acetate and butyrate. Reduced SCFAs are implicated in a range of conditions, including inflammatory bowel disease, arthritis, and allergies (11), with decreased maternal acetate levels in pregnancy linked to atopy, for example (133, 134). SCFAs also have links to DC functionality, impairing DC cytokine secretion and antigen presentation ability *in vitro* (135, 136). Urribe-Herranz et al. found vancomycin-mediated depletion of butyrate augmented the antitumour response in a murine radiotherapy model, through enhanced DC cross-presentation of tumour antigens (136). This suggests that antibiotics could be used to modulate DC antigen-presentation capacity via their impact on the gut microbiota, which could be advantageous in contexts such as cancer treatment or autoimmune disease. Conversely, reductions in SCFA caused by antibiotics could be mitigated through prebiotic or probiotic supplementation, to encourage growth of SCFA-generating species, or by direct supplementation with SCFAs.

In summary, we aimed to examine the impact of an antibiotics-induced dysbiosis on host immune homeostasis. Our findings reveal that while perturbation of the gut microbiota reduces levels of immunomodulatory SCFAs, the immune profiles in the spleen and MLNs remain largely unaffected under basal conditions. However, host cells may be programmed towards a greater immune response when challenged, suggesting a potential predisposition to inflammation. This study adds to the growing body of research highlighting the importance of understanding short- and long-term impacts of dysbiosis, particularly when triggered by antibiotics, and how it may set the stage for development of chronic diseases.

## Data Availability

The datasets presented in this study can be found in online repositories. The names of the repository/repositories and accession number(s) can be found below: https://www.ebi.ac.uk/ena, PRJEB79918.

## References

[1] Kennedy EA King KY Baldridge MT. Mouse microbiota models: comparing germ-free mice and antibiotics treatment as tools for modifying gut bacteria. Front Physiol. (2018) 9:1534. doi: 10.3389/fphys.2018.01534 30429801 PMC6220354

[2] Tan J Taitz J Sun SM Langford L Ni D Macia L. Your regulatory T cells are what you eat: how diet and gut microbiota affect regulatory T cell development. Front Nutr. (2022) 9. doi: 10.3389/fnut.2022.878382 PMC9067578 35529463

[3] Ivanov II Atarashi K Manel N Brodie EL Shima T Karaoz U. Induction of intestinal Th17 cells by segmented filamentous bacteria. Cell. (2009) 139:485–98. doi: 10.1016/j.cell.2009.09.033 PMC2796826 19836068

[4] Ekmekciu I von Klitzing E Fiebiger U Escher U Neumann C Bacher P. Immune responses to broad-spectrum antibiotic treatment and fecal microbiota transplantation in mice. Front Immunol. (2017) 8:397. doi: 10.3389/fimmu.2017.00397 28469619 PMC5395657

[5] Khosravi A Yáñez A Price JG Chow A Merad M Goodridge HS. Gut microbiota promote hematopoiesis to control bacterial infection. Cell Host Microbe. (2014) 15:374–81. doi: 10.1016/j.chom.2014.02.006 PMC4144825 24629343

[6] Ochoa-Repáraz J Mielcarz DW Ditrio LE Burroughs AR Foureau DM Haque-Begum S. Role of gut commensal microflora in the development of experimental autoimmune encephalomyelitis. J Immunol. (2009) 183:6041–50. doi: 10.4049/jimmunol.0900747 19841183

[7] Miyauchi E Shimokawa C Steimle A Desai MS Ohno H. The impact of the gut microbiome on extra-intestinal autoimmune diseases. Nat Rev Immunol. (2023) 23:9–23. doi: 10.1038/s41577-022-00727-y 35534624

[8] Carding S Verbeke K Vipond DT Corfe BM Owen LJ. Dysbiosis of the gut microbiota in disease. Microb Ecol Health Dis. (2015) 26:26191. doi: 10.3402/mehd.v26.26191 25651997 PMC4315779

[9] Wilkins LJ Monga M Miller AW. Defining dysbiosis for a cluster of chronic diseases. Sci Rep. (2019) 9:12918. doi: 10.1038/s41598-019-49452-y 31501492 PMC6733864

[10] Memon H Abdulla F Reljic T Alnuaimi S Serdarevic F Asimi ZV. Effects of combined treatment of probiotics and metformin in management of type 2 diabetes: A systematic review and meta-analysis. Diabetes Res Clin Pract. (2023) 202:110806. doi: 10.1016/j.diabres.2023.110806 37369280

[11] Tan J McKenzie C Potamitis M Thorburn AN Mackay CR Macia L. The role of short-chain fatty acids in health and disease. Adv Immunol. (2014) 121:91–119. doi: 10.1016/B978-0-12-800100-4.00003-9 24388214

[12] Ferrell JM Chiang JYL. Bile acid receptors and signaling crosstalk in the liver, gut and brain. Liver Research Bile Acids Metab liver Dis. (2021) 5:105–18. doi: 10.1016/j.livres.2021.07.002 PMC11791822 39957847

[13] Zhou L. Ahr function in lymphocytes: emerging concepts. Trends Immunol. (2016) 37:17–31. doi: 10.1016/j.it.2015.11.007 26700314 PMC4707131

[14] Franchi L Warner N Viani K Nuñez G. Function of nod-like receptors in microbial recognition and host defense. Immunol Rev. (2009) 227:106–28. doi: 10.1111/j.1600-065X.2008.00734.x PMC2679989 19120480

[15] Mogensen TH. Pathogen recognition and inflammatory signaling in innate immune defenses. Clin Microbiol Rev. (2009) 22:240–73. doi: 10.1128/CMR.00046-08 PMC2668232 19366914

[16] Hoerr V Zbytnuik L Leger C Tam PPC Kubes P Vogel HJ. Gram-negative and gram-positive bacterial infections give rise to a different metabolic response in a mouse model. J Proteome Res. (2012) 11:3231–45. doi: 10.1021/pr201274r PMC3368387 22483232

[17] Wang Q Li X Tang W Guan X Xiong Z Zhu Y. Differential gene sets profiling in gram-negative and gram-positive sepsis. Front Cell Infect Microbiol. (2022) 12:801232. doi: 10.3389/fcimb.2022.801232 35223539 PMC8863667

[18] Liu P Wu L Peng G Han Y Tang R Ge J. Altered microbiomes distinguish Alzheimer’s disease from amnestic mild cognitive impairment and health in a Chinese cohort. Brain Behav Immun. (2019) 80:633–43. doi: 10.1016/j.bbi.2019.05.008 31063846

[19] Magne F Gotteland M Gauthier L Zazueta A Pesoa S Navarrete P. The firmicutes/bacteroidetes ratio: A relevant marker of gut dysbiosis in obese patients? Nutrients. (2020) 12:1474. doi: 10.3390/nu12051474 32438689 PMC7285218

[20] Pushpanathan P Srikanth P Seshadri KG Selvarajan S Pitani RS Kumar TD. Gut microbiota in type 2 diabetes individuals and correlation with monocyte chemoattractant protein1 and interferon gamma from patients attending a tertiary care centre in chennai, India. Indian J Endocrinol Metab. (2016) 20:523–30. doi: 10.4103/2230-8210.183474 PMC4911843 27366720

[21] Salguero MV Al-Obaide MAI Singh R Siepmann T Vasylyeva TL. Dysbiosis of Gram-negative gut microbiota and the associated serum lipopolysaccharide exacerbates inflammation in type 2 diabetic patients with chronic kidney disease. Exp Ther Med. (2019) 18:3461–9. doi: 10.3892/etm.2019.7943 PMC6777309 31602221

[22] Patangia DV Anthony Ryan C Dempsey E Paul Ross R Stanton C. Impact of antibiotics on the human microbiome and consequences for host health. Microbiologyopen. (2022) 11:e1260. doi: 10.1002/mbo3.1260 35212478 PMC8756738

[23] Queen J Zhang J Sears CL. Oral antibiotic use and chronic disease: long-term health impact beyond antimicrobial resistance and Clostridioides difficile. Gut Microbes. (2020) 11:1092–103. doi: 10.1080/19490976.2019.1706425 PMC7524332 32037950

[24] Song W Tiruthani K Wang Y Shen L Hu M Dorosheva O. Trapping lipopolysaccharide to promote immunotherapy against colorectal cancer and attenuate liver metastasis. Adv Mater. (2018) 30:e1805007. doi: 10.1002/adma.201805007 30387230 PMC6580426

[25] Tirelle P Breton J Riou G Déchelotte P Coëffier M Ribet D. Comparison of different modes of antibiotic delivery on gut microbiota depletion efficiency and body composition in mouse. BMC Microbiol. (2020) 20:340. doi: 10.1186/s12866-020-02018-9 33176677 PMC7657353

[26] Joat N Bajagai YS Van TTH Stanley D Chousalkar K Moore RJ. The temporal fluctuations and development of faecal microbiota in commercial layer flocks. Anim Nutr. (2023) 15:197–209. doi: 10.1016/j.aninu.2023.07.006 38023383 PMC10679818

[27] Callahan B. RDP taxonomic training data formatted for DADA2 (RDP trainset 16/release 11.5). Zenodo (2017). doi: 10.5281/zenodo.801828.

[28] Tan J Ni D Taitz J Pinget GV Read M Senior A. Dietary protein increases T-cell-independent sIgA production through changes in gut microbiota-derived extracellular vesicles. Nat Commun. (2022) 13:4336. doi: 10.1038/s41467-022-31761-y 35896537 PMC9329401

[29] Usyk M Zolnik CP Patel H Levi MH Burk RD. Novel ITS1 fungal primers for characterization of the mycobiome. mSphere. (2017) 2:e00488–17. doi: 10.1128/mSphere.00488-17 PMC5729218 29242834

[30] Boutin RCT Sbihi H McLaughlin RJ Hahn AS Konwar KM Loo RS. Composition and associations of the infant gut fungal microbiota with environmental factors and childhood allergic outcomes. mBio. (2021) 12:e0339620. doi: 10.1128/mBio.03396-20 34060330 PMC8263004

[31] Browne AJ Chipeta MG Haines-Woodhouse G Kumaran EPA Hamadani BHK Zaraa S. Global antibiotic consumption and usage in humans 2000-18: a spatial modelling study. Lancet Planet Health. (2021) 5:e893–904. doi: 10.1016/S2542-5196(21)00280-1 PMC8654683 34774223

[32] Inklaar R de Jong H Bolt J van Zanden JL. Rebasing “Maddison”: new income comparisons and the shape of long-run economic development. Groningen Growth and Development Center. GGDC Res Memorandum. (2018) GD-174.

[33] Ni D Nanan R. Global and regional associations of enteric infections and asthma. medRxiv. (2024). doi: 10.1101/2024.05.21.24307671

[34] Ni D Nanan R. Global associations of maternal hypertensive disorders and offspring allergic disease burden. Clin Exp Allergy. (2024) 54:1024–6. doi: 10.1111/cea.14566 PMC11629057 39288820

[35] Diederich A. Generalized additive models. introduction R J Math Psychol. (2007) 51:339.

[36] Lane P Wood S Jones M Nelder J Lee Y Cortina-Borja M. Generalized additive models for location, scale and shape - Discussion. Appl Stat. (2005) 54:544–54.

[37] Verbeke T. Generalized additive models: an introduction with R by S. N. Wood. J R Stat Soc Ser A: Stat Soc. (2007) 170:262. doi: 10.1111/j.1467-985X.2006.00455_15.x

[38] Wood SN. Generalized additive models: an introduction with R. 2nd ed. New York: Chapman and Hall/CRC (2017).

[39] Akaike H. Information theory and an extension of the maximum likelihood principle. In: Parzen E Tanabe K Kitagawa G, editors. Selected papers of hirotugu akaike. Springer, New York, NY (1998). p. 199–213. doi: 10.1007/978-1-4612-1694-0_15

[40] Trimble MJ Mlynárčik P Kolář M Hancock REW. Polymyxin: alternative mechanisms of action and resistance. Cold Spring Harb Perspect Med. (2016) 6:a025288. doi: 10.1101/cshperspect.a025288 27503996 PMC5046685

[41] Avedissian SN Liu J Rhodes NJ Lee A Pais GM Hauser AR. A review of the clinical pharmacokinetics of polymyxin B. Antibiotics. (2019) 8:31. doi: 10.3390/antibiotics8010031 30909507 PMC6466567

[42] Eubank TA Hu C Gonzales-Luna AJ Garey KW. Detectable vancomycin stool concentrations in hospitalized patients with diarrhea given intravenous vancomycin. Pharmacoepidemiology. (2023) 2:283–8. doi: 10.3390/pharma2040024

[43] Falagas ME Kasiakou SK Saravolatz LD. Colistin: the revival of polymyxins for the management of multidrug-resistant gram-negative bacterial infections. Clin Infect Dis. (2005) 40:1333–41. doi: 10.1086/429323 15825037

[44] Gonzales M Pepin J Frost EH Carrier JC Sirard S Fortier L-C. Faecal pharmacokinetics of orally administered vancomycin in patients with suspected Clostridium difficile infection. BMC Infect Dis. (2010) 10:363. doi: 10.1186/1471-2334-10-363 21192802 PMC3022836

[45] Kaye KS Pogue JM Kaye D. 31 - polymyxins (Polymyxin B and colistin). In: Bennett JE Dolin R Blaser MJ, editors. Mandell, douglas, and bennett’s principles and practice of infectious diseases, Eighth Edition. W.B. Saunders, Philadelphia (2015). p. 401–405.e1. doi: 10.1016/B978-1-4557-4801-3.00031-X

[46] Liu M Chen M Yang Z. Design of amphotericin B oral formulation for antifungal therapy. Drug Delivery. (2017) 24:1–9. doi: 10.1080/10717544.2016.1225852 PMC8241147 28155335

[47] Pettit NN DePestel DD Fohl AL Eyler R Carver PL. Risk factors for systemic vancomycin exposure following administration of oral vancomycin for the treatment of clostridium difficile infection. Pharmacotherapy: J Hum Pharmacol Drug Ther. (2015) 35:119–26. doi: 10.1002/phar.1538 25689243

[48] Reikvam DH Erofeev A Sandvik A Grcic V Jahnsen FL Gaustad P. Depletion of murine intestinal microbiota: effects on gut mucosa and epithelial gene expression. PloS One. (2011) 6:e17996. doi: 10.1371/journal.pone.0017996 21445311 PMC3061881

[49] Thuny F Richet H Casalta J-P Angelakis E Habib G Raoult D. Vancomycin treatment of infective endocarditis is linked with recently acquired obesity. PloS One. (2010) 5:e9074. doi: 10.1371/journal.pone.0009074 20161775 PMC2818846

[50] Inoue Y Fukui H Xu X Ran Y Tomita T Oshima T. Colonic M1 macrophage is associated with the prolongation of gastrointestinal motility and obesity in mice treated with vancomycin. Mol Med Rep. (2019) 19:2591–8. doi: 10.3892/mmr.2019.9920 PMC6423659 30720127

[51] Zhang N Liu J Chen Z Chen N Gu F He Q. Integrated analysis of the alterations in gut microbiota and metabolites of mice induced after long-term intervention with different antibiotics. Front Microbiol. (2022) 13:832915. doi: 10.3389/fmicb.2022.832915 35847062 PMC9277126

[52] Xu X Fukui H Ran Y Tomita T Oshima T Watari J. Alteration of GLP-1/GPR43 expression and gastrointestinal motility in dysbiotic mice treated with vancomycin. Sci Rep. (2019) 9:4381. doi: 10.1038/s41598-019-40978-9 30867532 PMC6416360

[53] Sawaed J Zelik L Levin Y Feeney R Naama M Gordon A. Antibiotics damage the colonic mucus barrier in a microbiota-independent manner. Sci Adv. (2024) 10:eadp4119. doi: 10.1126/sciadv.adp4119 39259805 PMC11389797

[54] Sun L Zhang X Zhang Y Zheng K Xiang Q Chen N. Antibiotic-induced disruption of gut microbiota alters local metabolomes and immune responses. Front Cell Infect Microbiol. (2019) 9:99. doi: 10.3389/fcimb.2019.00099 31069173 PMC6491449

[55] Buchholz KR Reichelt M Johnson MC Robinson SJ Smith PA Rutherford ST. Potent activity of polymyxin B is associated with long-lived super-stoichiometric accumulation mediated by weak-affinity binding to lipid A. Nat Commun. (2024) 15:4733. doi: 10.1038/s41467-024-49200-5 38830951 PMC11148078

[56] Cullen TW Schofield WB Barry NA Putnam EE Rundell EA Trent MS. Antimicrobial peptide resistance mediates resilience of prominent gut commensals during inflammation. Science. (2015) 347:170–5. doi: 10.1126/science.1260580 PMC4388331 25574022

[57] Ingham HR Selkon JB Codd AA Hale JH. A study *in vitro* of the sensitivity to antibiotics of Bacteroides fragilis. J Clin Pathol. (1968) 21:432–6. doi: 10.1136/jcp.21.4.432 PMC473827 4301500

[58] Lagkouvardos I Joseph D Kapfhammer M Giritli S Horn M Haller D. IMNGS: A comprehensive open resource of processed 16S rRNA microbial profiles for ecology and diversity studies. Sci Rep. (2016) 6:33721. doi: 10.1038/srep33721 27659943 PMC5034312

[59] Pfeiffer N Desmarchelier C Blaut M Daniel H Haller D Clavel T. Acetatifactor muris gen. nov., sp. nov., a novel bacterium isolated from the intestine of an obese mouse. Arch Microbiol. (2012) 194:901–7. doi: 10.1007/s00203-012-0822-1 22659832

[60] Huffnagle GB Noverr MC. The emerging world of the fungal microbiome. Trends Microbiol. (2013) 21:334. doi: 10.1016/j.tim.2013.04.002 23685069 PMC3708484

[61] van Tilburg Bernardes E Pettersen VK Gutierrez MW Laforest-Lapointe I Jendzjowsky NG Cavin J-B. Intestinal fungi are causally implicated in microbiome assembly and immune development in mice. Nat Commun. (2020) 11:2577. doi: 10.1038/s41467-020-16431-1 32444671 PMC7244730

[62] Daïen CI Tan J Audo R Mielle J Quek LE Krycer JR. Gut-derived acetate promotes B10 cells with antiinflammatory effects. JCI Insight. (2021) 6:e144156. doi: 10.1172/jci.insight.144156 33729999 PMC8119207

[63] Fusco W Lorenzo MB Cintoni M Porcari S Rinninella E Kaitsas F. Short-chain fatty-acid-producing bacteria: key components of the human gut microbiota. Nutrients. (2023) 15:2211. doi: 10.3390/nu15092211 37432351 PMC10180739

[64] Fernández-Veledo S Vendrell J. Gut microbiota-derived succinate: Friend or foe in human metabolic diseases? Rev Endocr Metab Disord. (2019) 20:439–47. doi: 10.1007/s11154-019-09513-z PMC6938788 31654259

[65] Pinget GV Tan JK Ni D Taitz J Daien CI Mielle J. Dysbiosis in imiquimod-induced psoriasis alters gut immunity and exacerbates colitis development. Cell Rep. (2022) 40:111191. doi: 10.1016/j.celrep.2022.111191 35977500

[66] Takeuchi T Miyauchi E Kanaya T Kato T Nakanishi Y Watanabe T. Acetate differentially regulates IgA reactivity to commensal bacteria. Nature. (2021) 595:560–4. doi: 10.1038/s41586-021-03727-5 34262176

[67] Gupta S Basu S Bal V Rath S George A. Gut IgA abundance in adult life is a major determinant of resistance to dextran sodium sulfate-colitis and can compensate for the effects of inadequate maternal IgA received by neonates. Immunology. (2019) 158:19–34. doi: 10.1111/imm.13091 31215020 PMC6700465

[68] Salonen A Lahti L Salojärvi J Holtrop G Korpela K Duncan SH. Impact of diet and individual variation on intestinal microbiota composition and fermentation products in obese men. ISME J. (2014) 8:2218–30. doi: 10.1038/ismej.2014.63 PMC4992075 24763370

[69] Fang S Qin T Yu T Zhang G. Improvement of the gut microbiota *in vivo* by a short-chain fatty acids-producing strain lactococcus garvieae CF11. Processes. (2022) 10:604. doi: 10.3390/pr10030604

[70] Louis P Flint HJ. Formation of propionate and butyrate by the human colonic microbiota. Environ Microbiol. (2017) 19:29–41. doi: 10.1111/1462-2920.13589 27928878

[71] Scheithauer TPM Bakker GJ Winkelmeijer M Davids M Nieuwdorp M van Raalte DH. Compensatory intestinal immunoglobulin response after vancomycin treatment in humans. Gut Microbes. (2021) 13:1875109. doi: 10.1080/19490976.2021.1875109 33475461 PMC7833805

[72] Sun T Nguyen A Gommerman JL. Dendritic cell subsets in intestinal immunity and inflammation. J Immunol. (2020) 204:1075–83. doi: 10.4049/jimmunol.1900710 32071090

[73] Vatanen T Kostic AD d’Hennezel E Siljander H Franzosa EA Yassour M. Variation in microbiome LPS immunogenicity contributes to autoimmunity in humans. Cell. (2016) 165:842. doi: 10.1016/j.cell.2016.04.007 27133167 PMC4950857

[74] Hessle C Andersson B Wold AE. Gram-positive bacteria are potent inducers of monocytic interleukin-12 (IL-12) while gram-negative bacteria preferentially stimulate IL-10 production. Infect Immun. (2000) 68:3581–6. doi: 10.1128/IAI.68.6.3581-3586.2000 PMC97646 10816515

[75] Hessle CC Andersson B Wold AE. Gram-positive and Gram-negative bacteria elicit different patterns of pro-inflammatory cytokines in human monocytes. Cytokine. (2005) 30:311–8. doi: 10.1016/j.cyto.2004.05.008 15935951

[76] Karlsson H Larsson P Wold AE Rudin A. Pattern of Cytokine Responses to Gram-Positive and Gram-Negative Commensal Bacteria is Profoundly Changed when Monocytes Differentiate into Dendritic Cells. Scandinavian J Immunol. (2004) 59:628–8. doi: 10.1111/j.0300-9475.2004.01423at.x PMC387913 15102775

[77] Tietze K Dalpke A Morath S Mutters R Heeg K Nonnenmacher C. Differences in innate immune responses upon stimulation with gram-positive and gram-negative bacteria. J Periodontal Res. (2006) 41:447–54. doi: 10.1111/j.1600-0765.2006.00890.x 16953821

[78] Surbatovic M Popovic N Vojvodic D Milosevic I Acimovic G Stojicic M. Cytokine profile in severe Gram-positive and Gram-negative abdominal sepsis. Sci Rep. (2015) 5:11355. doi: 10.1038/srep11355 26079127 PMC4468818

[79] Cheng M Qian L Shen G Bian G Xu T Xu W. Microbiota modulate tumoral immune surveillance in lung through a γδT17 immune cell-dependent mechanism. Cancer Res. (2014) 74:4030–41. doi: 10.1158/0008-5472.CAN-13-2462 24947042

[80] Li F Hao X Chen Y Bai L Gao X Lian Z. The microbiota maintain homeostasis of liver-resident γδT-17 cells in a lipid antigen/CD1d-dependent manner. Nat Commun. (2017) 8:13839. doi: 10.1038/ncomms13839 PMC5227332 28067223

[81] Atarashi K Tanoue T Shima T Imaoka A Kuwahara T Momose Y. Induction of colonic regulatory T cells by indigenous Clostridium species. Science. (2011) 331:337–41. doi: 10.1126/science.1198469 PMC3969237 21205640

[82] Mazmanian SK Liu CH Tzianabos AO Kasper DL. An immunomodulatory molecule of symbiotic bacteria directs maturation of the host immune system. Cell. (2005) 122:107–18. doi: 10.1016/j.cell.2005.05.007 16009137

[83] Kamada N Hisamatsu T Okamoto S Chinen H Kobayashi T Sato T. Unique CD14 intestinal macrophages contribute to the pathogenesis of Crohn disease via IL-23/IFN-gamma axis. J Clin Invest. (2008) 118:2269–80. doi: 10.1172/JCI34610 PMC2391067 18497880

[84] Langer V Vivi E Regensburger D Winkler TH Waldner MJ Rath T. IFN-γ drives inflammatory bowel disease pathogenesis through VE-cadherin–directed vascular barrier disruption. J Clin Invest. (2019) 129:4691–707. doi: 10.1172/JCI124884 PMC6819119 31566580

[85] Monaco C Nanchahal J Taylor P Feldmann M. Anti-TNF therapy: past, present and future. Int Immunol. (2015) 27:55–62. doi: 10.1093/intimm/dxu102 25411043 PMC4279876

[86] Ran Y Fukui H Xu X Wang X Ebisutani N Tanaka Y. Alteration of colonic mucosal permeability during antibiotic-induced dysbiosis. Int J Mol Sci. (2020) 21:6108. doi: 10.3390/ijms21176108 32854266 PMC7504080

[87] Faye AS Allin KH Iversen AT Agrawal M Faith J Colombel J-F. Antibiotic use as a risk factor for inflammatory bowel disease across the ages: a population-based cohort study. Gut. (2023) 72:663–70. doi: 10.1136/gutjnl-2022-327845 PMC9998355 36623926

[88] Nguyen LH Örtqvist AK Cao Y Simon TG Roelstraete B Song M. Antibiotic use and the development of inflammatory bowel disease: a national case/control study in Sweden. Lancet Gastroenterol Hepatol. (2020) 5:986–95. doi: 10.1016/S2468-1253(20)30267-3 PMC8034612 32818437

[89] Theochari NA Stefanopoulos A Mylonas KS Economopoulos KP. Antibiotics exposure and risk of inflammatory bowel disease: a systematic review. Scandinavian J Gastroenterol. (2018) 53:1–7. doi: 10.1080/00365521.2017.1386711 29022402

[90] Skovbjerg S Martner A Hynsjö L Hessle C Olsen I Dewhirst FE. Gram-positive and gram-negative bacteria induce different patterns of cytokine production in human mononuclear cells irrespective of taxonomic relatedness. J Interferon Cytokine Res. (2010) 30:23–32. doi: 10.1089/jir.2009.0033 20028205

[91] Ivanov II de Llanos Frutos R Manel N Yoshinaga K Rifkin DB Sartor RB. Specific microbiota direct the differentiation of Th17 cells in the mucosa of the small intestine. Cell Host Microbe. (2008) 4:337–49. doi: 10.1016/j.chom.2008.09.009 PMC2597589 18854238

[92] Uddin MJ Thompson B Leslie JL Fishman C Sol-church K Kumar P. Investigating the impact of antibiotic-induced dysbiosis on protection from Clostridium difficile colitis by mouse colonic innate lymphoid cells. mBio. (2024) 15:e03338–23. doi: 10.1128/mbio.03338-23 PMC11209775 38376154

[93] Josefsdottir KS Baldridge MT Kadmon CS King KY. Antibiotics impair murine hematopoiesis by depleting the intestinal microbiota. Blood. (2017) 129:729–39. doi: 10.1182/blood-2016-03-708594 PMC5301822 27879260

[94] Hill DA Siracusa MC Abt MC Kim BS Kobuley D Kubo M. Commensal bacterial–derived signals regulate basophil hematopoiesis and allergic inflammation. Nat Med. (2012) 18:538–46. doi: 10.1038/nm.2657 PMC3321082 22447074

[95] Scott NA Andrusaite A Andersen P Lawson M Alcon-Giner C Leclaire C. Antibiotics induce sustained dysregulation of intestinal T cell immunity by perturbing macrophage homeostasis. Sci Transl Med. (2018) 10:eaao4755. doi: 10.1126/scitranslmed.aao4755 30355800 PMC6548564

[96] Baliga BS Sindel LJ Jenkins LD Sachen JB. Effect of polymyxin-B on T-lymphocyte protein synthesis. Biochem Biophys Res Commun. (1986) 135:649–54. doi: 10.1016/0006-291X(86)90042-2 3008731

[97] Bode C Muenster S Diedrich B Jahnert S Weisheit C Steinhagen F. Linezolid, vancomycin and daptomycin modulate cytokine production, Toll-like receptors and phagocytosis in a human *in vitro* model of sepsis. J Antibiot. (2015) 68:485–90. doi: 10.1038/ja.2015.18 PMC4579589 25735844

[98] Siedlar M Szczepanik A Wiȩckiewicz J Pituch-Noworolska A Zembala M. Vancomycin down-regulates lipopolysaccharide-induced tumour necrosis factor alpha (TNFα) production and TNFa-mRNA accumulation in human blood monocytes. Immunopharmacology. (1997) 35:265–71. doi: 10.1016/S0162-3109(96)00156-7 9043940

[99] Ogese MO Lister A Gardner J Meng X Alfirevic A Pirmohamed M. Deciphering adverse drug reactions: *in vitro* priming and characterization of vancomycin-specific T cells from healthy donors expressing HLA-A*32:01. Toxicol Sci. (2021) 183:139–53. doi: 10.1093/toxsci/kfab084 PMC8404995 34175955

[100] Bojang E Sheriff L Fu MS Wellings C Abdissa K Stavrou V. Vancomycin impairs macrophage fungal killing by disrupting mitochondrial morphology and function. bioRxiv. (2024). doi: 10.1101/2024.06.25.600580 PMC13251413 42053308

[101] Mesa-Arango AC Scorzoni L Zaragoza O. It only takes one to do many jobs: Amphotericin B as antifungal and immunomodulatory drug. Front Microbiol. (2012) 3:286. doi: 10.3389/fmicb.2012.00286 23024638 PMC3441194

[102] Drummond RA Desai JV Ricotta EE Swamydas M Deming C Conlan S. Long-term antibiotic exposure promotes mortality after systemic fungal infection by driving lymphocyte dysfunction and systemic escape of commensal bacteria. Cell Host Microbe. (2022) 30:1020–33.e6. doi: 10.1016/j.chom.2022.04.013 35568028 PMC9283303

[103] Lee Y Robbins N Cowen LE. Molecular mechanisms governing antifungal drug resistance. NPJ Antimicrob Resist. (2023) 1:5. doi: 10.1038/s44259-023-00007-2 38686214 PMC11057204

[104] O’Shaughnessy EM Lyman CA Walsh TJ. “Amphotericin B: Polyene Resistance Mechanisms.“ In Antimicrobial Drug Resistance: Mechanisms of Drug Resistance, edited by Douglas L. Mayers. (Totowa: Humana Press). (2009) pp. 295–305. doi: 10.1007/978-1-59745-180-2_25

[105] Atreya R Zimmer M Bartsch B Waldner MJ Atreya I Neumann H. Antibodies against tumor necrosis factor (TNF) induce T-cell apoptosis in patients with inflammatory bowel diseases via TNF receptor 2 and intestinal CD14^+^ macrophages. Gastroenterology. (2011) 141:2026–38. doi: 10.1053/j.gastro.2011.08.032 21875498

[106] Singh UP Singh NP Murphy EA Price RL Fayad R Nagarkatti M. Chemokine and cytokine levels in inflammatory bowel disease patients. Cytokine. (2016) 77:44–9. doi: 10.1016/j.cyto.2015.10.008 PMC4666758 26520877

[107] Tetta C Camussi G Modena V Di Vittorio C Baglioni C. Tumour necrosis factor in serum and synovial fluid of patients with active and severe rheumatoid arthritis. Ann Rheum Dis. (1990) 49:665–7. doi: 10.1136/ard.49.9.665 PMC1004199 1700672

[108] Kim D Zeng MY Núñez G. The interplay between host immune cells and gut microbiota in chronic inflammatory diseases. Exp Mol Med. (2017) 49:e339–9. doi: 10.1038/emm.2017.24 PMC5454439 28546562

[109] Mousa WK Chehadeh F Husband S. Microbial dysbiosis in the gut drives systemic autoimmune diseases. Front Immunol. (2022) 13:906258. doi: 10.3389/fimmu.2022.906258 36341463 PMC9632986

[110] Freuer D Linseisen J Meisinger C. Association between inflammatory bowel disease and both psoriasis and psoriatic arthritis. JAMA Dermatol. (2022) 158:1262–8. doi: 10.1001/jamadermatol.2022.3682 PMC9475439 36103169

[111] Li Y Guo J Cao Z Wu J. Causal association between inflammatory bowel disease and psoriasis: A two-sample bidirectional mendelian randomization study. Front Immunol. (2022) 13:916645. doi: 10.3389/fimmu.2022.916645 35757704 PMC9226443

[112] Fletcher JM Lalor SJ Sweeney CM Tubridy N Mills KHG. T cells in multiple sclerosis and experimental autoimmune encephalomyelitis. Clin Exp Immunol. (2010) 162:1–11. doi: 10.1111/j.1365-2249.2010.04143.x 20682002 PMC2990924

[113] Mitamura M Nakano N Yonekawa T Shan L Kaise T Kobayashi T. T cells are involved in the development of arthritis induced by anti-type II collagen antibody. Int Immunopharmacol. (2007) 7:1360–8. doi: 10.1016/j.intimp.2007.05.021 17673151

[114] Keenan JD Bailey RL West SK Arzika AM Hart J Weaver J. Azithromycin to reduce childhood mortality in sub-saharan africa. N Engl J Med. (2018) 378:1583–92. doi: 10.1056/NEJMoa1715474 PMC5849140 29694816

[115] Anthony WE Wang B Sukhum KV D’Souza AW Hink T Cass C. Acute and persistent effects of commonly used antibiotics on the gut microbiome and resistome in healthy adults. Cell Rep. (2022) 39:110649. doi: 10.10f16/j.celrep.2022.110649 35417701 PMC9066705

[116] Palleja A Mikkelsen KH Forslund SK Kashani A Allin KH Nielsen T. Recovery of gut microbiota of healthy adults following antibiotic exposure. Nat Microbiol. (2018) 3:1255–65. doi: 10.1038/s41564-018-0257-9 30349083

[117] Dessein R Bauduin M Grandjean T Le Guern R Figeac M Beury D. Antibiotic-related gut dysbiosis induces lung immunodepression and worsens lung infection in mice. Crit Care. (2020) 24:611. doi: 10.1186/s13054-020-03320-8 33076936 PMC7574210

[118] Elkrief A Méndez-Salazar EO Maillou J Vanderbilt CM Gogia P Desilets A. Antibiotics are associated with worse outcomes in lung cancer patients treated with chemotherapy and immunotherapy. NPJ Precis. Onc. (2024) 8:1–9. doi: 10.1038/s41698-024-00630-w PMC11252311 39014160

[119] McKee AM Kirkup BM Madgwick M Fowler WJ Price CA Dreger SA. Antibiotic-induced disturbances of the gut microbiota result in accelerated breast tumor growth. iScience. (2021) 24:103012. doi: 10.1016/j.isci.2021.103012 34522855 PMC8426205

[120] Winek K Engel O Koduah P Heimesaat MM Fischer A Bereswill S. Depletion of cultivatable gut microbiota by broad-spectrum antibiotic pretreatment worsens outcome after murine stroke. Stroke. (2016) 47:1354–63. doi: 10.1161/STROKEAHA.115.011800 PMC4839545 27056982

[121] Zhang X Borbet TC Fallegger A Wipperman MF Blaser MJ Müller A. An antibiotic-impacted microbiota compromises the development of colonic regulatory T cells and predisposes to dysregulated immune responses. mBio. (2021) 12:e03335–20. doi: 10.1128/mBio.03335-20 PMC7858066 33531385

[122] Balmer ML Schürch CM Saito Y Geuking MB Li H Cuenca M. Microbiota-derived compounds drive steady-state granulopoiesis via MyD88/TICAM signaling. J Immunol. (2014) 193:5273–83. doi: 10.4049/jimmunol.1400762 25305320

[123] Clarke TB Davis KM Lysenko ES Zhou AY Yu Y Weiser JN. Recognition of peptidoglycan from the microbiota by Nod1 enhances systemic innate immunity. Nat Med. (2010) 16:228–31. doi: 10.1038/nm.2087 PMC4497535 20081863

[124] Ganal SC Sanos SL Kallfass C Oberle K Johner C Kirschning C. Priming of natural killer cells by nonmucosal mononuclear phagocytes requires instructive signals from commensal microbiota. Immunity. (2012) 37:171–86. doi: 10.1016/j.immuni.2012.05.020 22749822

[125] Thackray LB Handley SA Gorman MJ Poddar S Bagadia P Briseño CG. Oral antibiotic treatment of mice exacerbates the disease severity of multiple flavivirus infections. Cell Rep. (2018) 22:3440–3453.e6. doi: 10.1016/j.celrep.2018.03.001 29590614 PMC5908250

[126] Vanderkerken M Baptista AP De Giovanni M Fukuyama S Browaeys R Scott CL. ILC3s control splenic cDC homeostasis via lymphotoxin signaling. J Exp Med. (2021) 218:e20190835. doi: 10.1084/jem.20190835 33724364 PMC7970251

[127] Sikder MAA Rashid RB Ahmed T Sebina I Howard DR Ullah MA. Maternal diet modulates the infant microbiome and intestinal Flt3L necessary for dendritic cell development and immunity to respiratory infection. Immunity. (2023) 56:1098–1114.e10. doi: 10.1016/j.immuni.2023.03.002 37003256

[128] Diehl GE Longman RS Zhang J-X Breart B Galan C Cuesta A. Microbiota restricts trafficking of bacteria to mesenteric lymph nodes by CX3CR1hi cells. Nature. (2013) 494:116–20. doi: 10.1038/nature11809 PMC3711636 23334413

[129] Lynn DJ Benson SC Lynn MA Pulendran B. Modulation of immune responses to vaccination by the microbiota: implications and potential mechanisms. Nat Rev Immunol. (2022) 22:33–46. doi: 10.1038/s41577-021-00554-7 34002068 PMC8127454

[130] Lamousé-Smith ES Tzeng A Starnbach MN. The intestinal flora is required to support antibody responses to systemic immunization in infant and germ free mice. PloS One. (2011) 6:e27662. doi: 10.1371/journal.pone.0027662 22114681 PMC3219679

[131] Oh JZ Ravindran R Chassaing B Carvalho FA Maddur MS Bower M. TLR5-mediated sensing of gut microbiota is necessary for antibody responses to seasonal influenza vaccination. Immunity. (2014) 41:478–92. doi: 10.1016/j.immuni.2014.08.009 PMC4169736 25220212

[132] Hong S-H. Influence of microbiota on vaccine effectiveness: “Is the microbiota the key to vaccine-induced responses? J Microbiol. (2023) 61:483–94. doi: 10.1007/s12275-023-00044-6 PMC10098251 37052795

[133] Lee-Sarwar KA Kelly RS Lasky-Su J Zeiger RS O’Connor GT Sandel MT. Fecal short-chain fatty acids in pregnancy and offspring asthma and allergic outcomes. J Allergy Clin Immunol Pract. (2020) 8:1100–1102.e13. doi: 10.1016/j.jaip.2019.08.036 31494295 PMC7056490

[134] Thorburn AN McKenzie CI Shen S Stanley D Macia L Mason LJ. Evidence that asthma is a developmental origin disease influenced by maternal diet and bacterial metabolites. Nat Commun. (2015) 6:7320. doi: 10.1038/ncomms8320 26102221

[135] Nastasi C Candela M Bonefeld CM Geisler C Hansen M Krejsgaard T. The effect of short-chain fatty acids on human monocyte-derived dendritic cells. Sci Rep. (2015) 5:16148. doi: 10.1038/srep16148 26541096 PMC4635422

[136] Uribe-Herranz M Rafail S Beghi S Gil-de-Gómez L Verginadis I Bittinger K. Gut microbiota modulate dendritic cell antigen presentation and radiotherapy-induced antitumor immune response. J Clin Invest. (2020) 130:466–79. doi: 10.1172/JCI124332 PMC6934221 31815742

