# Supplementary material for: Antibiotic-mediated dysbiosis leads to activation of inflammatory pathways
Source: Front Immunol. 2025 Jan 9;15:1493991. doi: 10.3389/fimmu.2024.1493991 (PMC11754057; doi:10.3389/fimmu.2024.1493991)
Supplement: Supplementary file 1 [file DataSheet1.zip › Supplementary_Material.docx]

Supplementary Material

# Supplementary Tables and Figures

**Supplementary Tables**

Table S1-2 are GAM estimates and statistical outputs. The model estimates and related standard errors (Std.Error) were shown for parametric terms. For non-parametric smooth terms, their estimated and reference degrees of freedom (i.e. edf, sumEDF, Ref.df) and their test statistics were shown.

**Table S1.** Relative fit of GAM analyzing the predictors for age-standardized inflammatory bowel disease (IBD) incidence rate (IBD%) globally. AIC = Akaike information criterion. sumEDF indicates the degrees of freedom of the models. Model 16 was selected as the best model because of the lowest AIC. (related to Figure 6)

| **GAM** | **AIC** | **sumEDF** | **Formula** |
| --- | --- | --- | --- |
| 0 | 5535.093 | 164.91 | gam(IBD% ~ 1 + s(Country, bs = "re")) |
| 1 | 5484.476 | 169.33 | gam(IBD% ~ s(Country, bs = "re") + s(GDP)) |
| 2 | 5483.403 | 167.47 | gam(IBD% ~ s(Country, bs = "re") + s(Year)) |
| 3 | 5466.237 | 171.63 | gam(IBD% ~ s(Year) + s(GDP) + s(Country, bs = "re")) |
| 4 | 5352.781 | 175.84 | gam(IBD% ~ te(Year, GDP) + s(Country, bs = "re")) |
| 5 | 5481.126 | 172.77 | gam(IBD% ~ s(ANTIBIOTIC.CONSUMPTION) + s(Country, bs = "re")) |
| 6 | 5449.299 | 175.51 | gam(IBD% ~ s(ANTIBIOTIC.CONSUMPTION) + s(Year) + s(Country, bs = "re")) |
| 7 | 5461.934 | 177.17 | gam(IBD% ~ s(ANTIBIOTIC.CONSUMPTION) + s(GDP) + s(Country, bs = "re")) |
| 8 | 5436.978 | 179.59 | gam(IBD% ~ s(ANTIBIOTIC.CONSUMPTION) + s(Year) + s(GDP) + s(Country, bs = "re")) |
| 9 | 5354.420 | 176.85 | gam(IBD% ~ s(ANTIBIOTIC.CONSUMPTION) + te(Year, GDP) + s(Country, bs = "re")) |
| 10 | 5444.488 | 180.15 | gam(IBD% ~ te(ANTIBIOTIC.CONSUMPTION, GDP) + s(Year) + s(Country, bs = "re")) |
| 11 | 5433.675 | 191.48 | gam(IBD% ~ te(ANTIBIOTIC.CONSUMPTION, Year) + s(GDP) + s(Country, bs = "re")) |
| 12 | 5071.345 | 221.43 | gam(IBD% ~ te(ANTIBIOTIC.CONSUMPTION, Year, GDP) + s(Country, bs = "re")) |
| 13 | 5445.612 | 186.89 | gam(IBD% ~ te(ANTIBIOTIC.CONSUMPTION, Year) + s(Country, bs = "re")) |
| 14 | 5471.002 | 176.61 | gam(IBD% ~ te(ANTIBIOTIC.CONSUMPTION, GDP) + s(Country, bs = "re")) |
| 15 | 5096.472 | 232.23 | gam(IBD% ~ s(ANTIBIOTIC.CONSUMPTION) + s(Year) + s(GDP) + ti(ANTIBIOTIC.CONSUMPTION, Year, GDP) + s(Country, bs = "re"), data = complete |
| **16** | **4966.310** | **252.65** | **gam(IBD% ~ s(ANTIBIOTIC.CONSUMPTION) + s(Year) + s(GDP) + ti(ANTIBIOTIC.CONSUMPTION, Year, GDP) + ti(ANTIBIOTIC.CONSUMPTION, Year) + ti(ANTIBIOTIC.CONSUMPTION, GDP) + ti(GDP, Year) + s(Country, bs = "re"))** |

**Table S2.** Estimated effects of antibiotic consumption (ANTIBIOTIC.CONSUMPTION), GDP per capita and year on IBD% globally. (related to Figure 6)

| **Parametric coefficients** | | | | |
| --- | --- | --- | --- | --- |
|  | **Estimate** | **Std.Error** | **t value** | **Pr(>\|t\|)** |
| **(Intercept)** | 4.467 | 285.901 | 0.016 | 0.988 |
| **Approximate significance of smooth terms** | | | | |
|  | **edf** | **Ref.df** | **F** | **P value** |
| **s(ANTIBIOTIC.CONSUMPTION)** | 7.952 | 8.660 | 8.925 | <2e-16 |
| **s(Year)** | 3.899 | 4.979 | 7.317 | 1.03e-06 |
| **s(GDP)** | 8.456 | 8.900 | 8.553 | <2e-16 |
| **ti(ANTIBIOTIC.CONSUMPTION, Year, GDP)** | 38.034 | 42.235 | 7.547 | <2e-16 |
| **ti(ANTIBIOTIC.CONSUMPTION, Year)** | 10.113 | 11.277 | 5.086 | <2e-16 |
| **ti(ANTIBIOTIC.CONSUMPTION, GDP)** | 9.508 | 10.727 | 8.478 | <2e-16 |
| **ti(Year, GDP)** | 9.685 | 11.106 | 8.920 | <2e-16 |
| **s(Country)** | 164.000 | 35.000 | 4326.229 | <2e-16 |
| R-sq.(adj) = 0.991 Deviance explained = 99.2% | | | | |
| GCV = 0.28722 Scale est. = 0.26408 n = 3135 | | | | |


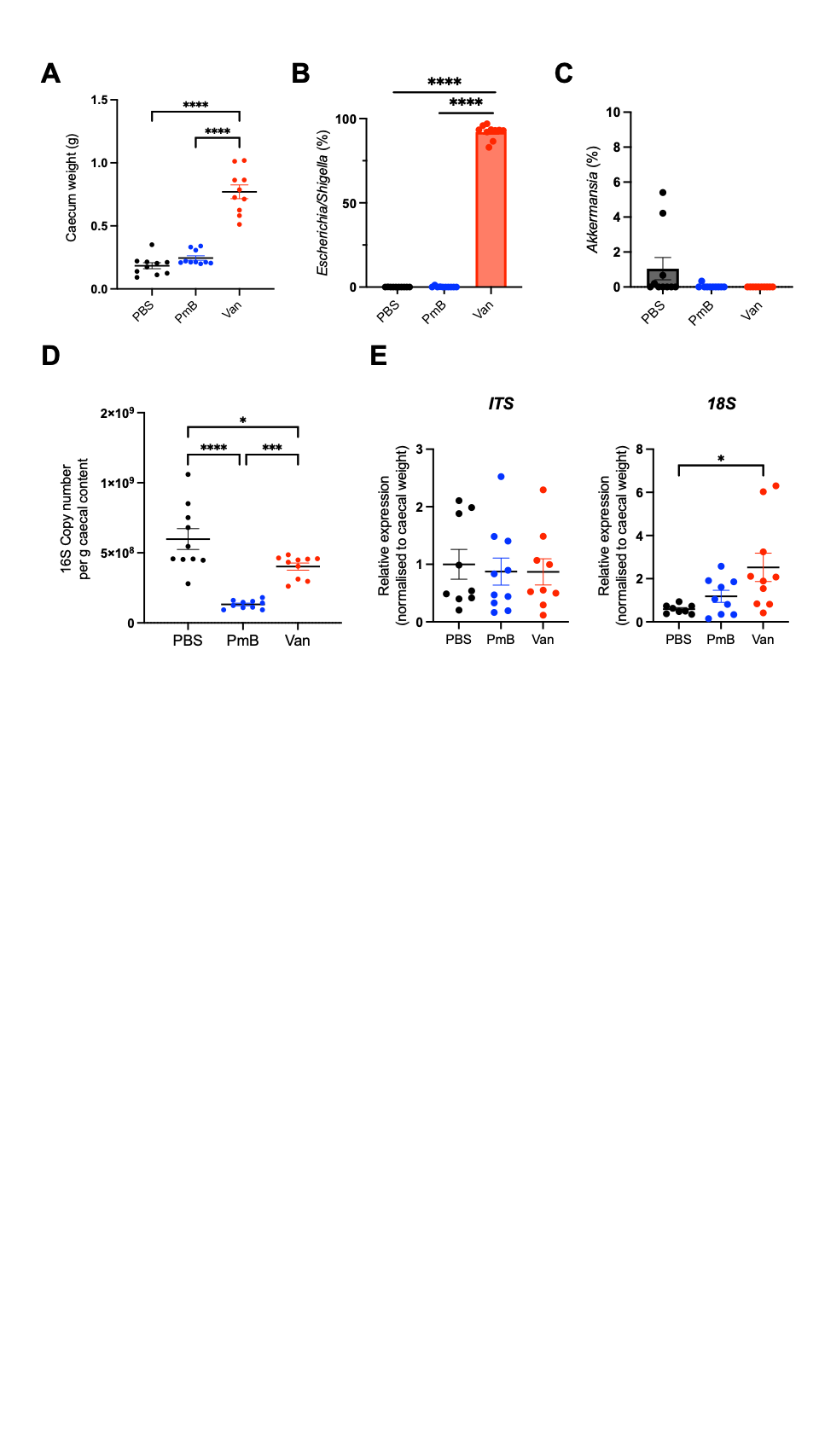


**Supplementary Figure 1.** (**A**) Total weight of caecum. (**B-C**) Differential abundance analysis of *Escherichia/Shigella* and *Akkermansia*, as assessed using ALDEx2, with expected Benjamini-Hochberg corrected p-value of Wilxocon test, with ****p<0.0001 (**D**) 16S copy number per g caecal conent, as determined by qPCR. (**E)** Relative expression of *ITS* and *18S* rRNA sequences per g caecal content, as determined by qPCR. Data are represented as mean ± SEM with *p<0.05, **<0.01, ***0.001, ****<0.0001 by ordinary one-way ANOVA followed by Tukey’s multiple comparisons test.


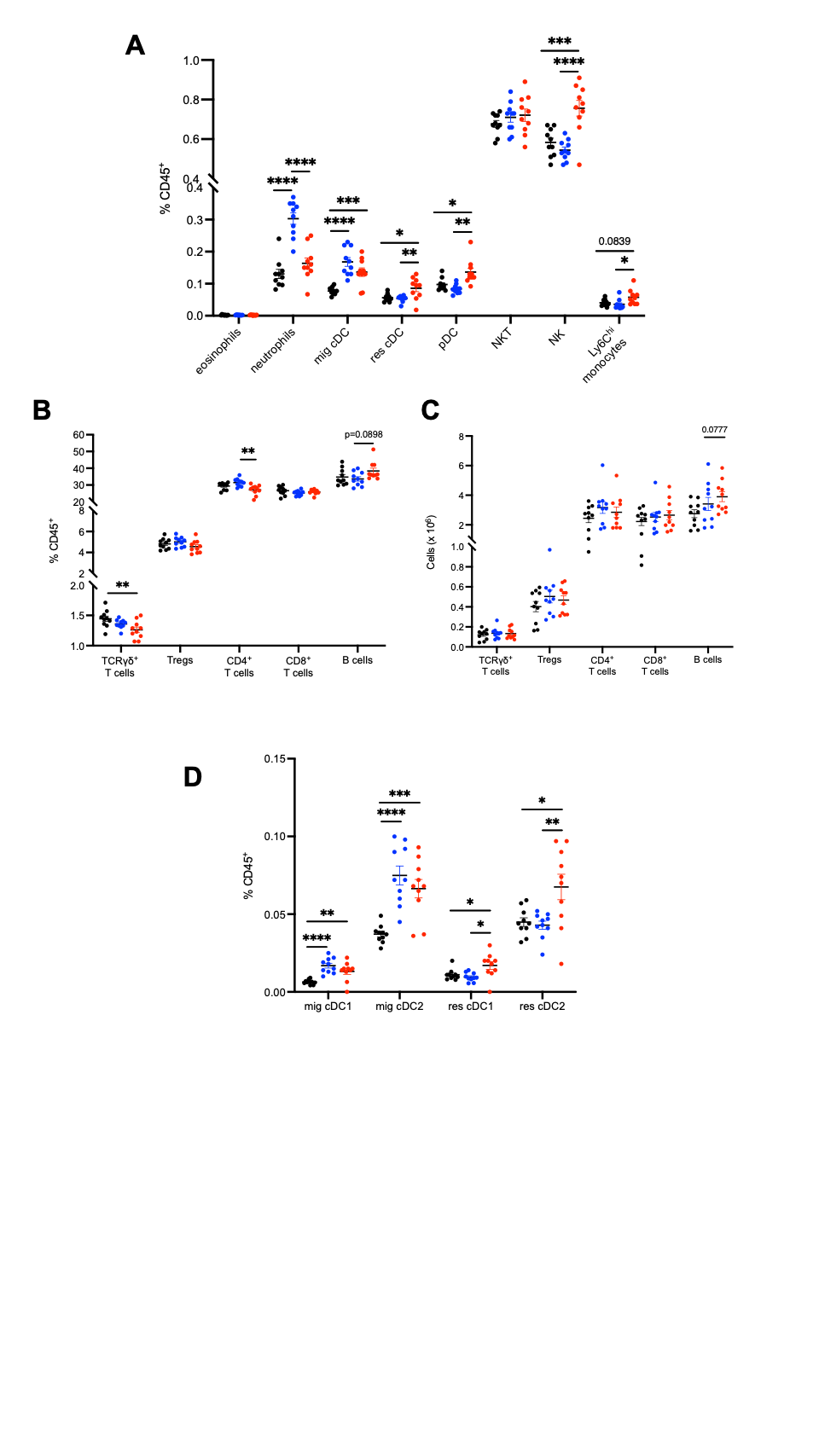


**Supplementary Figure** **2.** (**A**) Proportion of eosinophils, neutrophils, migratory conventional dendritic cells (mig cDC), resident cDCs (res cDC), plasmacytoid dendritic cells (pDC), natural killer T (NKT) cells, natural killer (NK) cells and LyC6^hi^ monocytes as quantified by flow cytometry (**B**) Proportions of TCRγδ^+^ T cells, regulatory T cells (Tregs), CD4^+^  and CD8^+^ T cells, and B cells in the MLNs as well as (**C**) total numbers of these cells. (**D**) Proportions of cDC1 and cDC2 subsets.

**
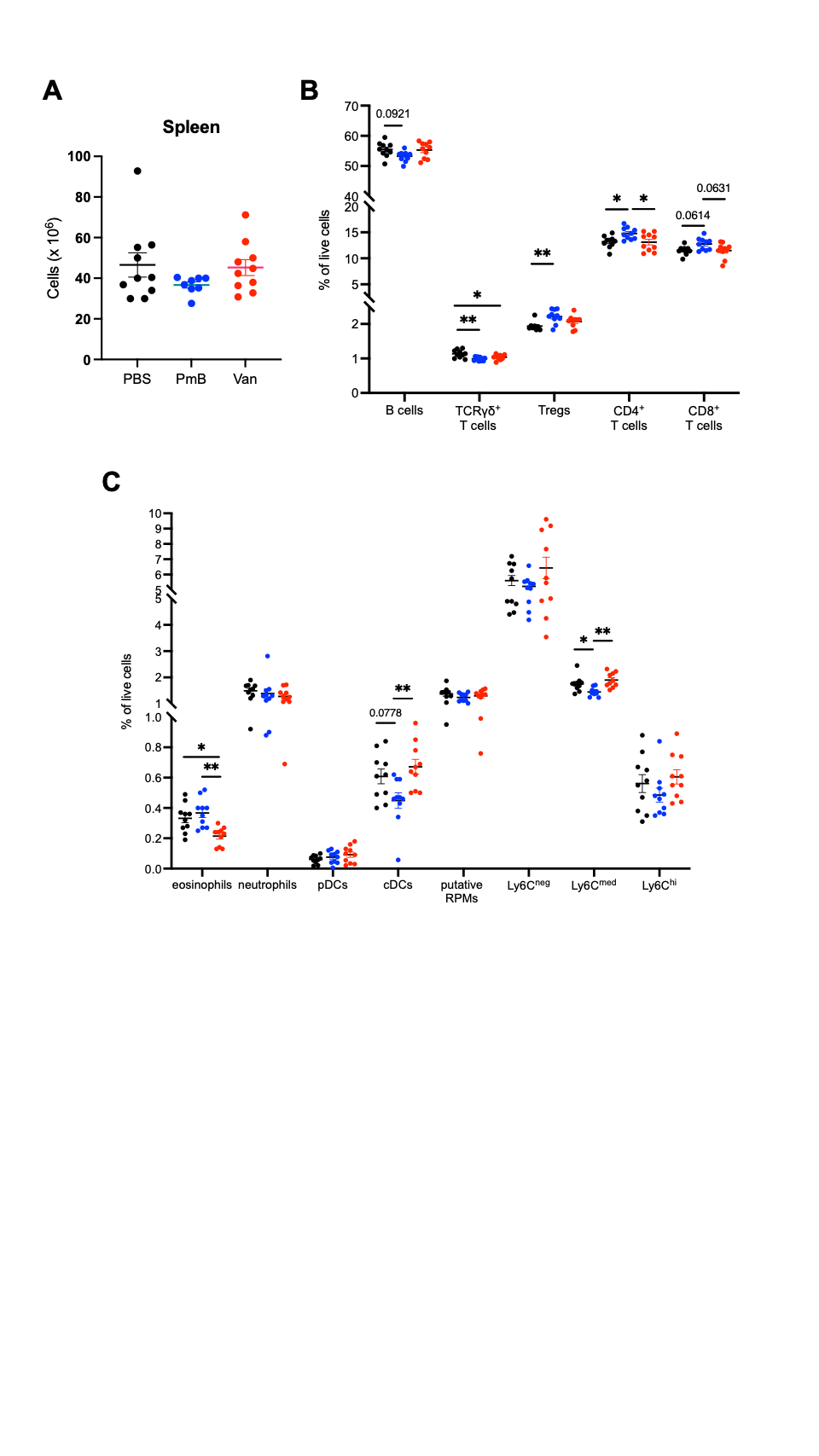
**

**Supplementary Figure 3.** (**A**) Total cellularity in spleen. (**B**) Proportion (of live cells) of B cells, TCRγδ^+^ T cells, regulatory T cells (Tregs), CD4^+^ and CD8^+^ T cells. (**C**) Proportion of live cells of eosinophils, neutrophils, plasmacytoid dendritic cells (pDCs), conventional dendritic cells (cDCs), putative red pulp macrophages (RPMs), and monocyte subsets (Ly6C^hi,^ Ly6C^mid^ Ly6C^neg^) as quantified by flow cytometry.

**
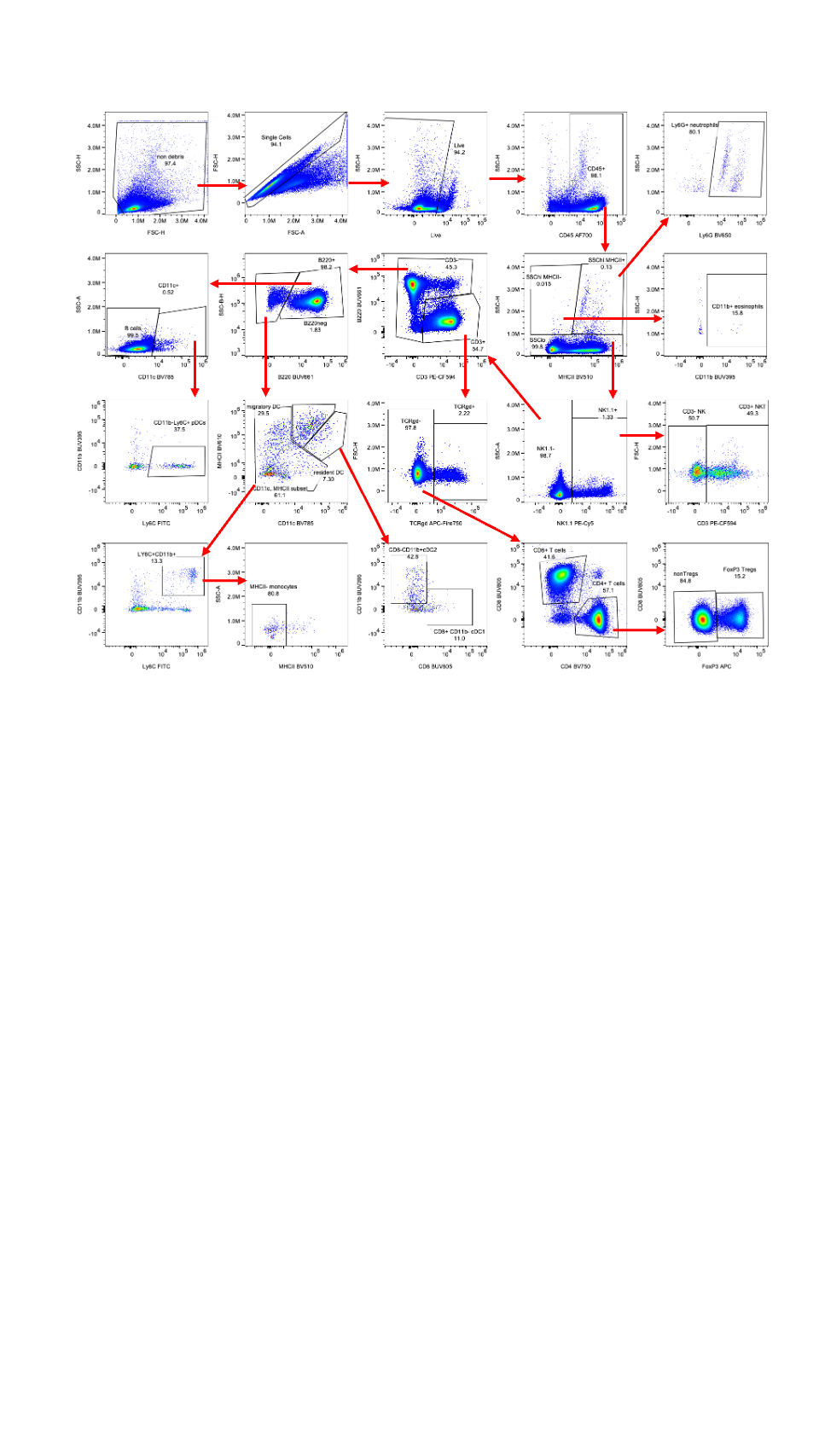
**

**Supplementary Figure 4.** Gating strategy for analysis of MLN immune cell profile, as in Figure 3.

**
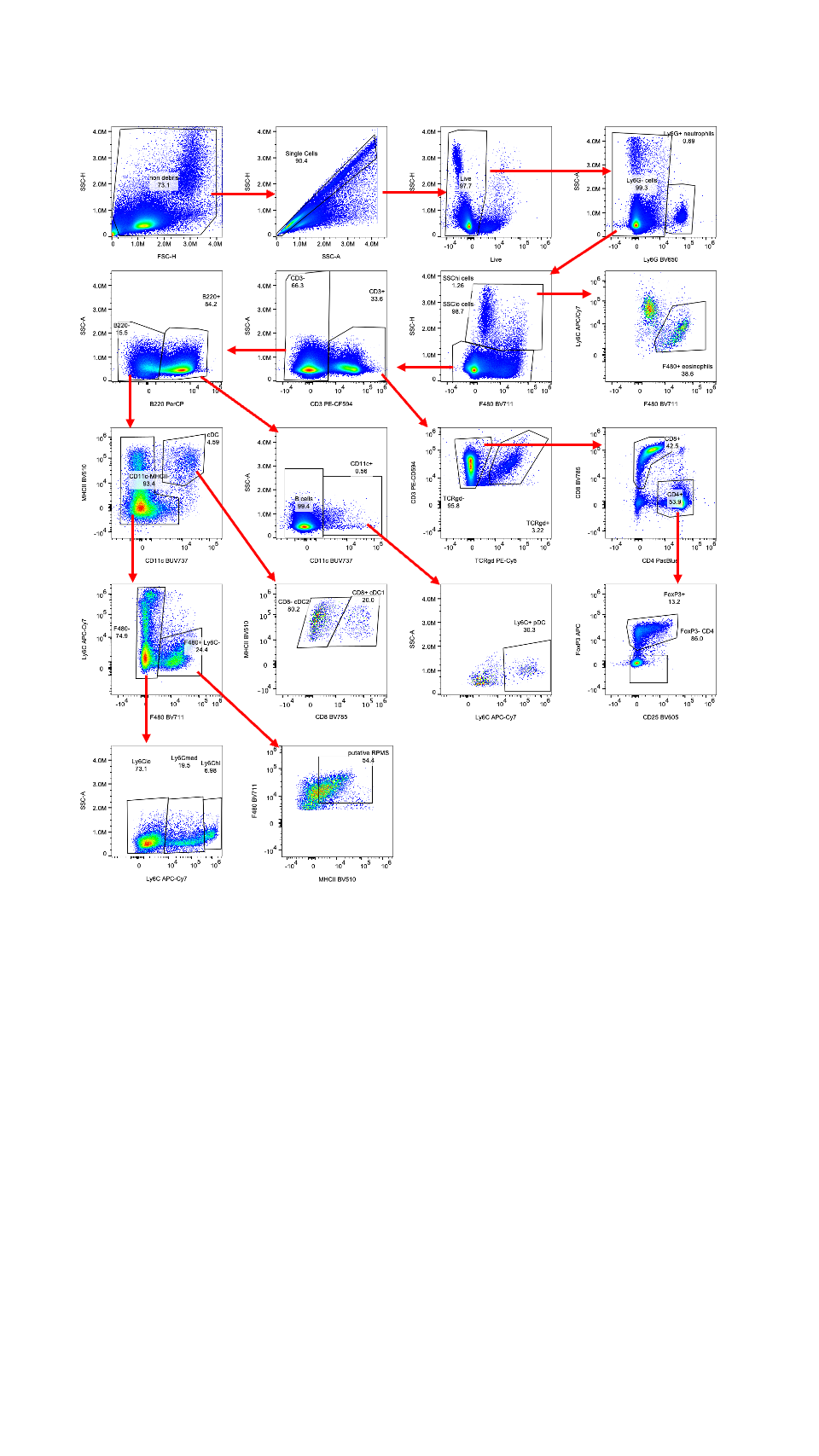
**

**Supplementary Figure 5.** Gating strategy for analysis of spleen immune cell profile, as in Figure 4


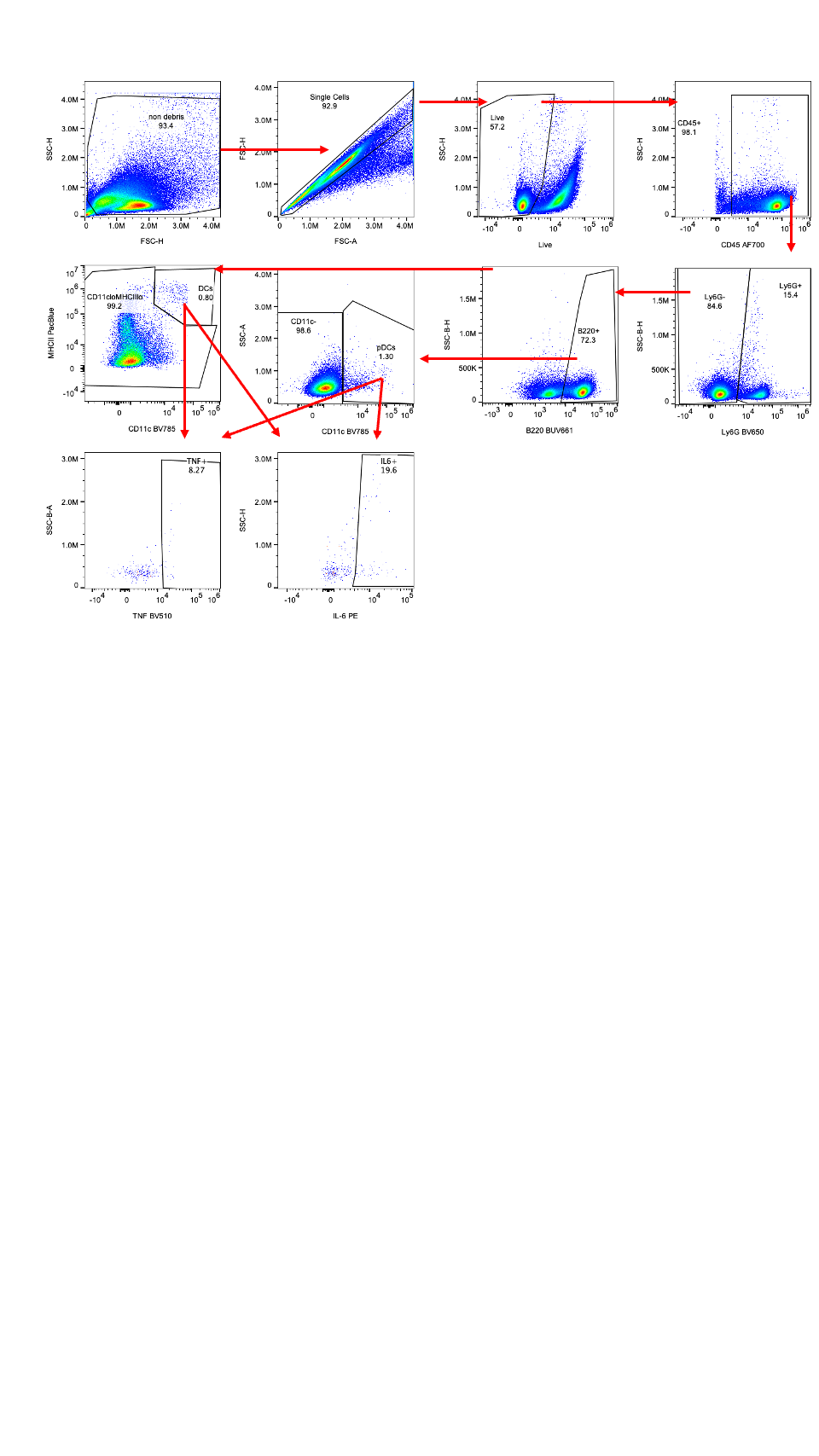


**Supplementary Figure 6**. Gating strategy for analysis of LPS stimulated splenocytes, as in Figure 4


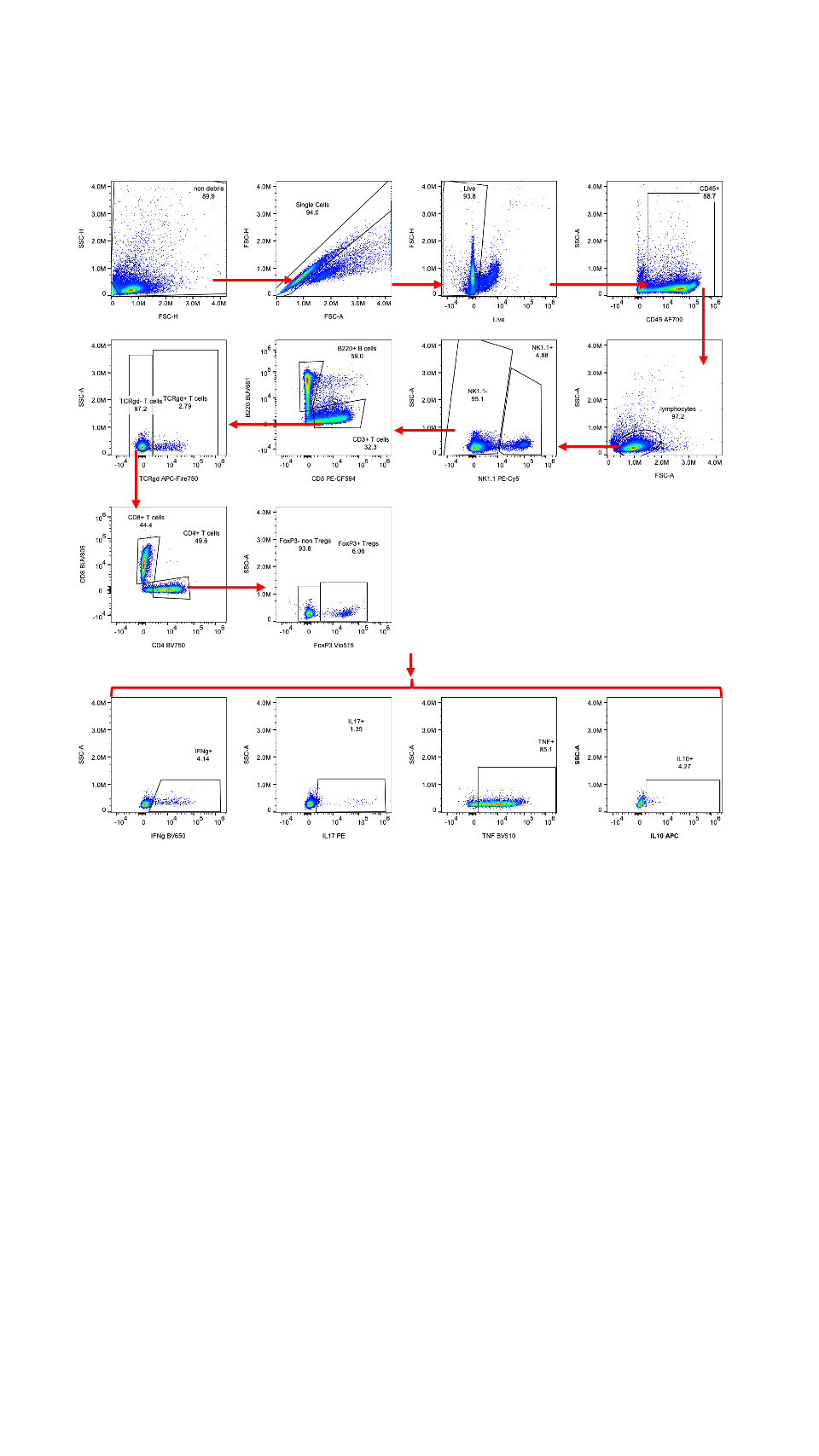


**Supplementary Figure 7.** Gating strategy for analysis of PMA/ionomycin stimulated splenocytes, as in Figure 5.

**
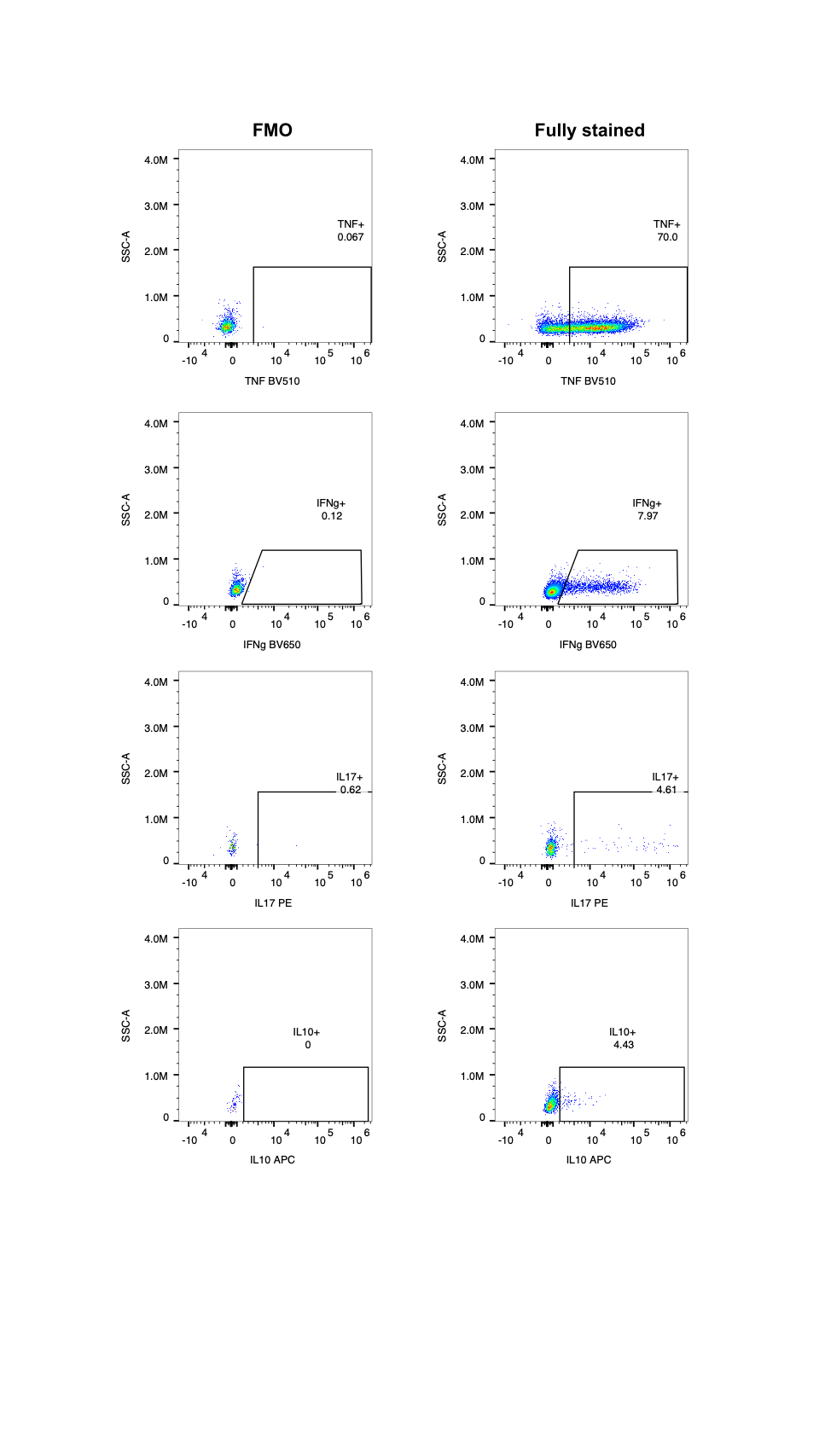
**

**Supplementary Figure 8:** Representative flow cytometry plots showing fluorescence minus one (FMO) controls and fully stained samples used to define the gating strategy in Supplementary Figure 7, for Figure 5 of Results section.


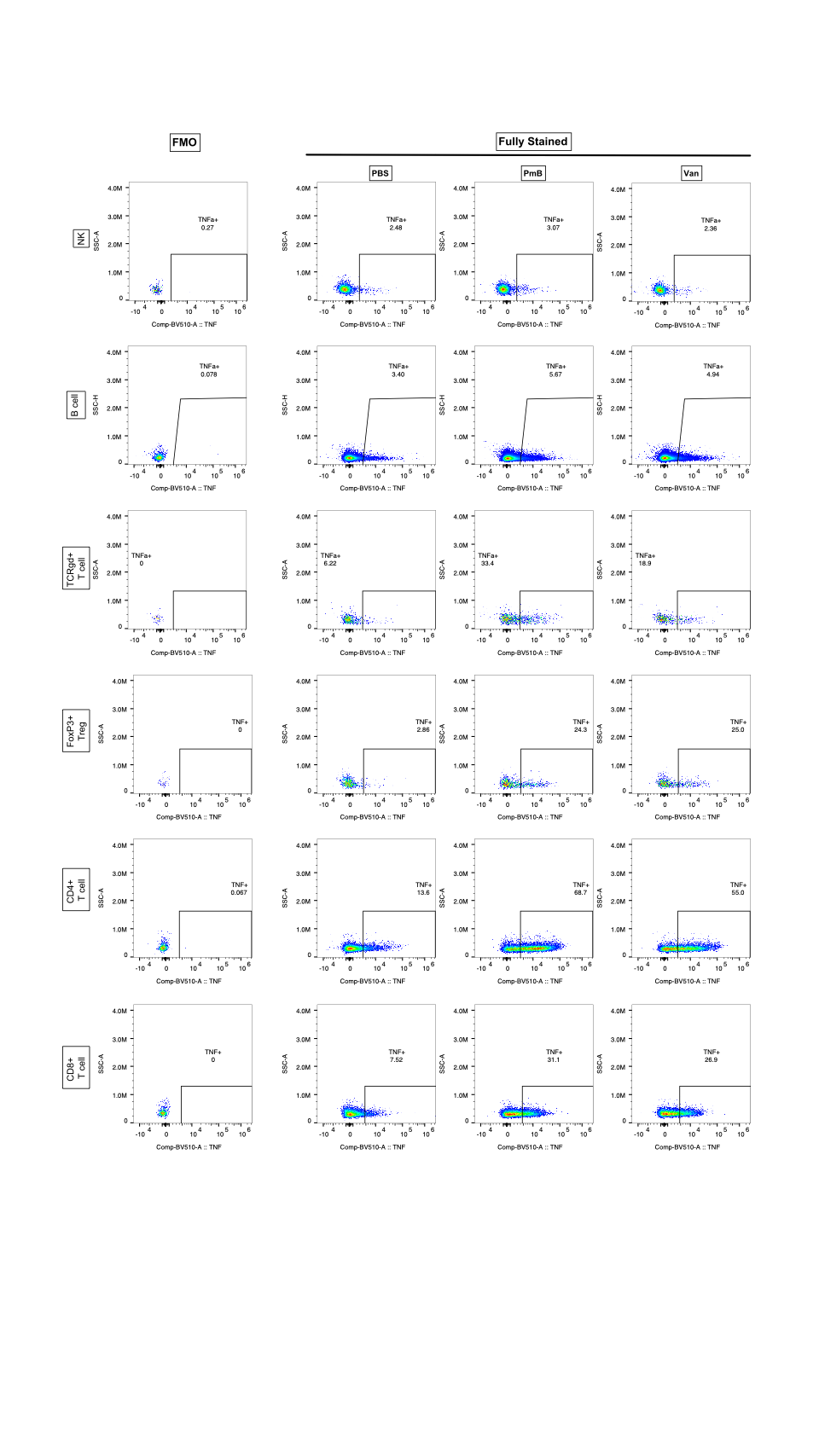


**Supplementary Figure 9**: Representative flow cytometry plots showing FMO control and fully stained samples for TNF^+^ cells in subsets presented in Figure 5 of Results.


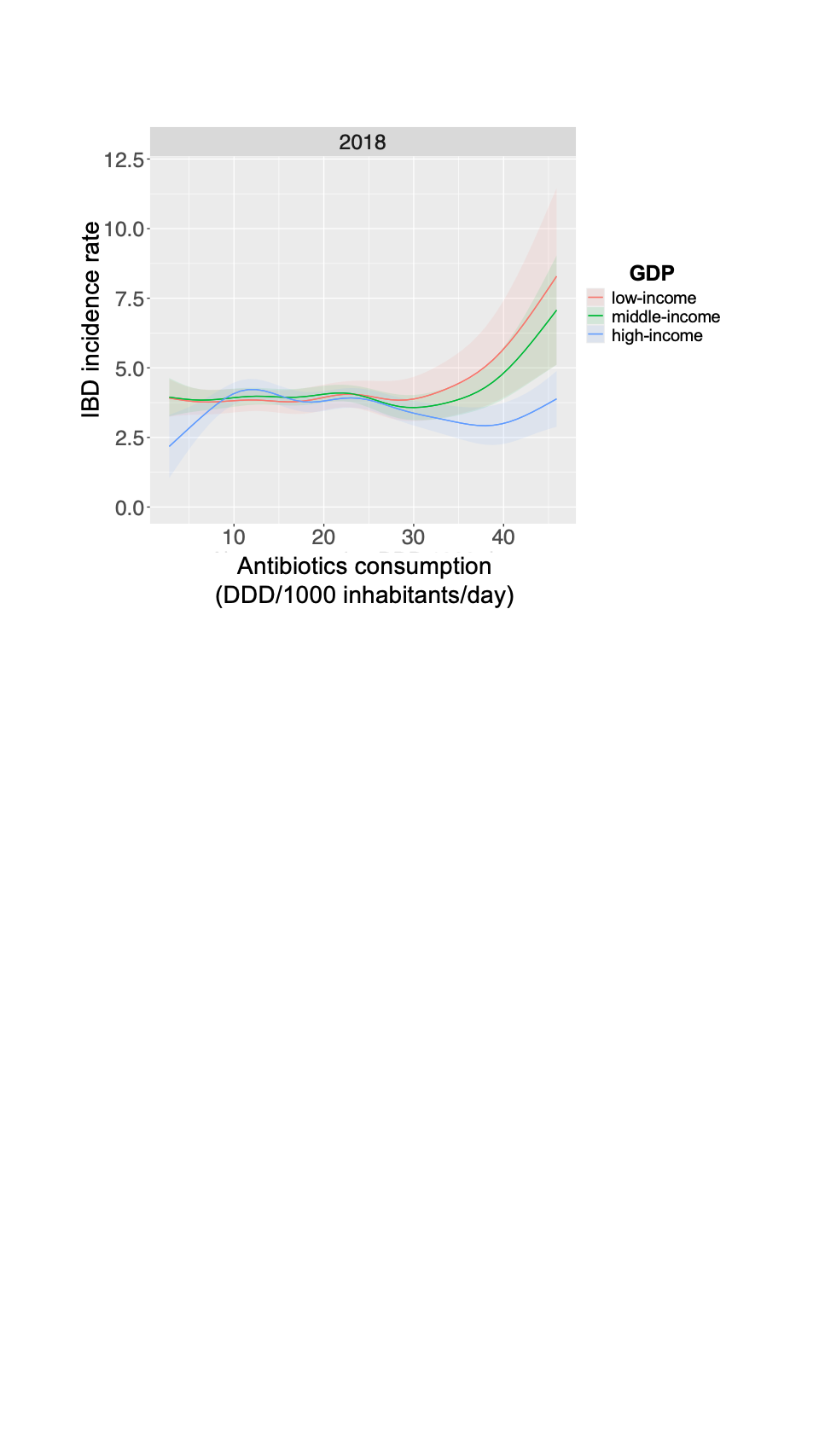


**Supplementary Figure 10**. Modelled effects of antibiotic consumption on global inflammatory bowel disease (IBD) disease burden (incidence rate) stratified by 25% (red, low-income), 50% (green, median-income) and 75% (blue, high-income) quantiles of gross domestic product (GDP).
